# Knockout of *OsRbohD* (the NADPH Oxidase Gene) Enhances Saline-Alkaline Stress Tolerance and Grain Yield in Rice by Reducing ROS Accumulation

**DOI:** 10.1186/s12284-026-00928-2

**Published:** 2026-06-23

**Authors:** Xiaolong Liu, Ping Ji, Xingjie Li, Hongtao Yang, Bo Peng, Kai Jiang, Chen Xu, Chang-Jie Jiang, Nan Dai, Shunlan Wan, Bingxi Wu, Si Chen, Wenbin Liao, Zhipeng Quan

**Affiliations:** 1 School of Life Sciences and Environmental Resources, Yichun University, Yichun, 336000 Jiangxi China; 2 Institute of Agricultural Resources and Environment. Jilin Academy of Agriculture Sciences, Changchun, 130033 Jilin China; 3 Shandong Rice Research Institute, Shandong Academy of Agricultural Sciences, Jinan, 250100 Shandong China; 4 College of Agriculture, Jiangxi Agricultural University, Nanchang, 330045 Jiangxi China

**Keywords:** Grain yield, *OsRbohD* knockout, Reactive oxygen species (ROS), Rice (*Oryza sativa* L.), Saline‒alkaline (SA) stress

## Abstract

Saline-alkaline (SA) stress, characterized by high salinity and alkalinity, severely constrains global crop productivity by inducing excessive accumulation of reactive oxygen species (ROS). This study revealed that CRISPR/Cas9-mediated knockout of the NADPH oxidase gene *OsRbohD* (*OsRbohD*-KO) significantly enhanced SA stress tolerance in rice. Compared with wild-type plants, the *OsRbohD*-KO lines exhibited a marked reduction in ROS accumulation in seeds, leaves, and roots under both saline and alkaline stress. This resulted in significantly improved seed germination, bud development, and root elongation. Physiologically, the *OsRbohD*-KO seedlings maintained higher leaf survival, chlorophyll content and K^+^ content, while showing reduced malondialdehyde (MDA) levels, membrane injury and Na^+^ accumulation. In severely SA soils, *OsRbohD*-KO increased seedling emergence by 5.77–28.85%, plant growth by 1.51–23.64%, root growth by 6.80–23.66%, and grain yield by 12.53–175.23%, driven by improvements in spikelets per panicle, grain filling, and 1000-grain weight. Molecular analysis revealed that *OsRbohD*-KO upregulated the expression of stress-related genes, including a cell death inhibitor gene (*OsBI-1)*, Na^+^ and K^+^ transporter-related genes (*OsHKT1*, *OsHKT7* and *OsPEX11*), proline biosynthesis-related genes (*OsP5CS1* and *OsP5CS2*), and a cell protection and stress signal transduction-related gene (*OsJRL*), and downregulated the expression of the cell death gene *OsKOD-1*. Conversely, *OsRbohD* overexpression lines showed increased sensitivity to SA stress. Our results suggest that suppressing *OsRbohD* is a highly effective strategy for alleviating SA damage and improving rice productivity in marginal environments.

**Supplementary Information:**

The online version contains supplementary material available at https://doi.org/10.1186/s12284-026-00928-2.

## Introduction

Soil salinization and alkalization are agricultural and environmental disasters that cause severe crop losses worldwide (Wang et al. 2016a; Wei et al. 2017). Owing to the high salinity and alkalinity in this soil, plants grown in saline-alkaline (SA) soils suffer from osmotic stress, high ion toxicity and high-pH stress (Liu et al. 2022b; Jiang et al. 2023). Rice (*Oryza sativa* L.) is sensitive to SA stress (Munns and Tester, 2008). SA stress can suppress rice growth by directly inhibiting plant growth (Lv et al. 2013; Wei et al. 2015) and impairing root growth (Feng et al. 2016; Liu et al. 2021), resulting in an imbalance in physiological metabolism (Zhang et al. 2017), a decrease in photosynthetic efficiency (Ma et al. 2022), and ultimately severe yield loss (Liu et al. 2022b; Qi et al. 2024). Rice grown in SA paddy soils suffers from severe growth inhibition and yield loss, with yields reaching only 12.9–90.7% of the global average (Seck et al. 2012). Previous studies have shown that residual sodium carbonate (Na_2_CO_3_) in SA paddy soils during the reproductive stage is the main cause of reduced rice yields (Wang et al. 2018). Rice plants cultivated in SA soils exhibited more severe damage under high alkalinity stress, leading to severe root damage and rice yields ranging from only 0.7 to 4.9 t ha^− 1^ (Seck et al. 2012; Wang et al. 2018). Thus, increasing the tolerance of rice to SA stress, especially high-pH stress, remains a significant challenge for stabilizing grain yield worldwide.

Reactive oxygen species (ROS) serve as crucial signaling molecules in plants and mediate diverse physiological processes, including growth regulation and responses to environmental stress factors (Li and Kim 2021). Under normal physiological conditions, ROS levels in plant cells are maintained in a dynamically balanced state through the coordinated action of ROS-producing and ROS-scavenging systems (Choudhury et al. 2017). However, various environmental factors, such as salinity, alkalinity, high temperature, and drought, disrupt this balance, triggering excessive ROS accumulation (Choudhury et al. 2017; Liu et al. 2022a, 2025). Excessive ROS accumulation can induce severe oxidative stress in plants, leading to damage to cellular components, including membranes (via lipid peroxidation), proteins, RNA and DNA, which is an important mechanism underlying the detrimental effects of SA stress on rice (Zhang et al. 2017; Liu et al. 2019). Recent studies have linked the inhibitory effects of SA stress on rice growth to the overaccumulation of ROS in roots (Zhang et al. 2017). SA stress resulted in the excessive accumulation of O_2_•^-^ and H_2_O_2_ in rice roots, causing a significant increase in malondialdehyde (MDA) levels and membrane injury (Liu et al. 2019). Furthermore, exogenous application of paraquat (a chemical inducer of ROS formation) caused severe damage to rice seedlings because of the overaccumulation of ROS (Liu et al. 2019), whereas supplementation with antioxidants and certain plant hormones (e.g., ABA) effectively reduced ROS levels and alleviated root damage and leaf withering (Liu et al. 2019, 2022b; Feng et al. 2024). Collectively, these findings underscore the pivotal role of inhibiting excessive ROS accumulation to enable rice to cope with SA stress.

ROS homeostasis is regulated by the balance between ROS generation and the antioxidant defense system (Liu et al. 2020), which indicates that enhancing antioxidant defense capacity or suppressing ROS production may mitigate stress-reduced ROS overaccumulation (Guan et al. 2017; Liu et al. 2022a, 2023b). However, severe environmental stress overwhelms the antioxidant defense system, leading to irreversible cellular damage and plant death (Sun et al. 2019). Therefore, targeted inhibition of excessive ROS production represents a promising approach for mitigating ROS overaccumulation (Kerchev et al. 2015; Fang et al. 2021). In plants, ROS are generated primarily via plasma membrane nicotinamide adenine dinucleotide phosphate (NADPH) oxidases, also known as respiratory burst oxidase homologs (Rbohs) (Sagi and Fluhr 2006). Rboh family genes play essential roles in regulating plant growth and stress responses (Nestler et al. 2014; Wang et al. 2023). In rice, nine Rboh family genes have been shown to encode Rbohs (Liu et al. 2012; Wang et al. 2013), and accumulating evidence indicates their involvement in regulating plant growth and response to stress factors (Shi et al. 2020; Wang et al. 2016b; Chen et al. 2024; Meng et al. 2025), which highlights the critical role of Rboh-mediated ROS regulation in plant stress adaptation. We previously demonstrated that *OsRbohB* (Os09g0438000) plays a pivotal role in rice response to heat stress by regulating ROS generation (Liu et al. 2023a), and CRISPR/Cas9-mediated knockout of *OsRbohB* reduces ROS overaccumulation and enhances heat stress tolerance in rice (Liu et al. 2025). These findings collectively indicate that *OsRboh* family gene-mediated ROS production is a key regulatory node in rice response to environmental stresses.

The *RbohD* gene is widely implicated in the regulation of plant growth, development, and stress responses across plant species. *AtRbohD* was constitutively expressed in all organs of *Arabidopsis thaliana* and was responsible for extracellular ROS production during bacterial-induced stomatal closure (Torres et al. 2002). In rice, *OsRbohD* exhibits prominent expression in calli, leaves, panicles, and roots (Liu et al. 2013). The *OsRbohD* protein contains conserved functional domains, including NADPH-ribose-binding and NADPH-adenine-binding sites, which are critical for its ROS-producing activity (Yoshiaki et al. 2005). *OsRbohD* is induced by *OsRACK1* to promote H_2_O_2_ production, thereby regulating rice seed germination (Zhang et al. 2014). Additionally, *OsRbohD*-mediated NADPH accumulation triggered ROS bursts and contributed to *Pijx*-regulated resistance against *Magnaporthe oryzae* in rice (Xiao et al. 2024). These results provide a robust theoretical foundation for further investigations of the regulatory function of *OsRbohD* in mediating SA stress tolerance in rice.

This study aimed to elucidate the role of *OsRbohD* in rice under SA stress and to evaluate whether targeted knockout of *OsRbohD* using CRISPR/Cas9 could increase SA tolerance by reducing ROS accumulation. We show that the knockout of *OsRbohD* significantly reduces ROS-mediated damage, thereby improving the growth, development and physiological metabolism of rice at different stages, and ultimately increasing the grain yield of rice under SA stress conditions. These findings provide an effective genetic strategy for improving the SA stress tolerance and productivity of rice plants grown in SA soils.

## Materials and Methods

### Plant Materials

The japonica rice variety Nipponbare (*Oryza sativa* subsp. *japonica cv. Nipponbare*) was used as the wild-type (WT) plant in all the experiments. The sequence of *OsRbohD* (Gene ID: Os05g0528000, Accession: AK120905, Yoshiaki et al. 2005) was retrieved from the National Center for Biotechnology Information (NCBI) database. CRISPR/Cas9-mediated mutagenesis of *OsRbohD* was performed using a vector system as previously described (Ma et al. 2015; Liu et al. 2025). Target sites within the open reading frame (ORF) of *OsRbohD* (Fig. S1a) were designed using the web-based tool CRISPR-GE/Target Design (http://skl.scau.edu.cn/) (Xie et al. 2017), following the target design principles outlined by He et al. (2021). Two single guide RNAs (sgRNAs) were designed to target the first and second exons of *OsRbohD* (Liu et al. 2017), with target sequences of 5’-GCATTGCGGGAAATCGTCGC-3’ and 5’-AGCTGTTCGACACCTTGAGC-3’, respectively. The construction of the *OsRbohD* CRISPR/Cas9 knockout vector followed the protocol described by Ma et al. (2015). Chimeric primers containing the target sequences (Table S1) were used for overlapping PCR to generate single sgRNA expression cassettes. The integrated sgRNA expression cassettes were amplified via nested PCR using site-specific primers (Pps and Pgs) and then ligated into the empty plasmid using Golden Gate ligation to construct the CRISPR/Cas9 knockout vector (Engler et al.^ 2008^; Ma et al. 2015). The recombinant vector was used to generate *OsRbohD*-knockout (*OsRbohD*-KO) plants by *Agrobacterium*-mediated transformation, which was performed by Edgene Biotechnology Co., Ltd. (Wuhan, China) (Dong et al. 2024; Zhao et al. 2024).

To construct the *OsRbohD*-overexpressing (*OsRbohD*-OE) vectors, the ubiquitin (ubi) promoter and NOS terminator sequences were cloned and inserted into the pCAMBIA1300 vector to generate the recombinant vector pCAMBIA1300-ubi. The full-length ORF of *OsRbohD* was amplified using gene-specific primers (Table S1) and inserted into pCAMBIA1300-ubi via Golden Gate ligation to generate the pCAMBIA1300-ubi-*OsRbohD* overexpression vector (Fig. S1b) (Zhang et al. 2022; Liu et al. 2025). The *OsRbohD* gene, driven by the ubiquitin promoter, was introduced into WT rice using *Agrobacterium tumefaciens* strain EHA105, following the method described by Toki et al. (2006). Transformation of the *OsRbohD*-OE plants was also conducted by Edgene Biotechnology Co., Ltd. (Wuhan, China) (Dong et al. 2024; Zhao et al. 2024).

Twenty *OsRbohD*-KO lines and twenty *OsRbohD*-OE lines were initially identified through hygromycin B resistance assays (Fig. S2a, b). Primary transformants (T0 plants) were self-pollinated to produce T1 generation seeds, as described previously (Liu et al. 2022b, 2025). T1 progenies were screened using genomic DNA analysis (Fig. S2c), standard PCR for the genotyping assay (Fig. S2d), hygromycin B resistance testing (Fig. S2e), and Cas9 detection testing (Fig. S2f) and then selfed to obtain homozygous T2 seeds (Liu et al. 2022b). Quantitative real-time PCR (qRT‒PCR) was performed to verify the transcriptional levels of *OsRbohD* in different lines compared with those in WT plants (Fig. S3) using primers designed with Primer 5.0 software outside the gene editing region (Table S1). Two *OsRbohD*-OE lines (OE-1 and OE-3) with significant overexpression and two *OsRbohD*-KO lines (KO-7 and KO-15) with distinct base deletions or insertions from different calli were selected for subsequent experiments.

### Plant Growth Conditions

#### Saline and Alkaline Stress at the Germination Stage

After surface sterilization, 30 seeds from the different rice lines were germinated in a Petri dish under different treatments, as described previously (Liu et al. 2025). Four saline stress treatments (50, 100, 150, and 200 mmol/L NaCl, designated SS1, SS2, SS3, and SS4, respectively) and four alkaline stress treatments (20, 40, 60, and 80 mmol/L Na_2_CO_3_, designated AS1, AS2, AS3, and AS4, respectively) were applied to different rice lines, with distilled water (pH = 6.95) used as the control (CK1). All the treatments were performed in a controlled growth chamber (SPT-P150C; Shanghai CIMO Medical Instrument Co., Ltd., China) in the dark at 30 °C, with five biological replicates. The germination energy and germination rate were measured on the 3rd and 7th days of germination, respectively, following the methods described by Lv et al. (2013). Germination energy refers to the percentage of germinated seeds within a specified time (usually on the 3rd day after sowing) (Lv et al. 2013) and was calculated using the following formula: (number of germinated seeds on the 3rd day/total number of seeds) × 100%. Owing to the severe inhibition of roots induced by alkaline stress (Lv et al. 2013; Feng et al. 2016), the rice seeds were considered to have germinated when the bud length was equal to half the length of the seed in this study, according to the methods described by Lv et al. (2013) and Liu et al. (2025). The bud length, mean root length and contents of O_2_•^-^ and H_2_O_2_ in the rice seeds of different lines were measured on the 7th day of germination.

### Saline and Alkaline Stress at the Seedling Stage

Seeds from different lines were grown to the three-leaf stage in a controlled growth chamber (28 °C day/22°C night, 12-h photoperiod, and 350 mmol photons m^− 2^ s^− 1^ light intensity) (Liu et al. 2022b, 2025). The seedlings were then transferred to distilled water (CK2), saline-stress (40, 80, 120 mmol/L NaCl, designated M-SS1, M-SS2, M-SS3), or alkaline-stress (10, 15, 20 mmol/L Na_2_CO_3_, designated M-AS1, M-AS2, M-AS3) conditions, with five biological replicates, as described in previous studies (Lv et al. 2013; Zhang et al. 2017; Liu et al. 2022b). The seedling growth status was photographed and measured on the 3rd and 7th days of treatment. The withered leaf rates were measured at the 3rd and 7th day during the treatments, being recorded as 1 if the whole leaf was dry and brown and as 0.5 if half of the leaf was dry and brown (Liu et al. 2022b, 2025). On the 7th day, the chlorophyll content in leaves and the contents of O_2_•^-^ and H_2_O_2_ in leaves and roots were measured. Re-watering was conducted after 6 days of SA stress, and seedlings growth status, leaf withering rate and chlorophyll content was measured in different lines after 24 h of re-watering.

### Saline-Alkaline Soil Treatments at the Seedling Stage

Surface-sterilized seeds were germinated uniformly, and 30 germinated seeds per line were sown in plastic cups (8 cm height, 6 cm internal diameter) containing 120 g of SA soil with different pH values. Different degrees of SA soils were prepared by mixing normal paddy soil and severely alkaline soil according to the weight ratio, as previously described (Liu et al. 2022b). The mixing weight ratios of normal paddy soil to severely alkaline soil (W: W) were as follows: 10:0 (CK3, pH: 7.53), 9:1 (YS1, pH: 8.11), 8:2 (YS2, pH: 8.52), 7:3 (YS3, pH: 8.84), 6:4 (YS4, pH: 9.10), 5:5 (YS5, pH: 9.33), 4:6 (YS6, pH: 9.49), 3:7 (YS7, pH: 9.78), 2:8 (YS8, pH: 10.01), and 1:9 (YS9, pH: 10.27). The EC values of the different soils were as follows: 104.5, 291.7, 397.0, 602.7, 761.0, 1028.0, 1203.7, 1463.3, 1716.0, 1838.0 and 1981.7 µS/cm (CK-YS9 treatments). All the cups were placed in a controlled growth chamber (SPT-P150C; Shanghai CIMO Medical Instrument Co., Ltd., China) under conditions of 28 °C day/22°C night, 12-h photoperiod, and 350 mmol photons m^− 2^ s^− 1^ light intensity, with five biological replicates. Equal amounts of water were added regularly to maintain soil moisture. The seedling growth status was photographed on the 10th day, and the emergence rate, shoot length, shoot fresh weight, and root fresh weight were measured.

### Saline-Alkaline Soil Treatments Throughout the Entire Growth Stage

Germinated seeds were sown in pots filled with normal paddy soil (Liu et al. 2022b). Approximately thirty-day-old seedlings were transplanted into pots containing 4 kg of soil with three SA stress levels: moderate (pH 8.52, YS2), medium (pH 9.33, YS5) and severe (pH 9.78, YS7), and normal paddy soil (pH 7.53) as the control (CK3). Each pot contained two clumps of three seedlings per line, and all the pots were placed in a controlled phytotron and managed using traditional cultivation methods, as described previously (Liu et al. 2025), with five biological replicates. The plant growth status, panicle phenotype, and root growth were photographed at the tillering, heading, filling, and mature stages. The plant growth, root growth and yield component indices were measured at the mature stage.

### Rboh Inhibitor Treatments

In this study, diphenylene iodonium (DPI) and p-chloromercuribenzoate (PCMB), which are inhibitors of plant NADPH oxidase (Gestelen et al. 1997; Glyan’ko and Ischenko 2010), were used to evaluate the role of Rboh in the tolerance of rice to SA stress. DPI inhibited the accumulation of H_2_O_2_ in potato tuber tissues under fungal elicitor treatment (Yoshioka et al. 2001), whereas PCMB (a thiol reagent) completely inhibited plant NADPH oxidase activity at a concentration of 60 µM (Gestelen et al. 1997).

Rice seedlings (cv. Nipponbare) at approximately the three-leaf stage were subjected to the following treatments: deionized water (CK), alkaline stress (15 mmol/L Na_2_CO_3_), DPI (5, 10, 20, 30, 50 µmol/L) under non-stress or alkaline stress conditions, and PCMB (1, 2, 5, 10, 15 µmol/L) under non-stress or alkaline stress conditions. The seedling growth status was photographed on the 7th day, and withered leaf rate, chlorophyll content, O_2_•^-^ content, and H_2_O_2_ content were measured.

### Treatments for *OsRbohD* Expression Pattern Analysis

Rice seedlings (cv. Nipponbare) at approximately the three-leaf stage were treated with deionized water (CK), saline stress (120 mmol/L NaCl, SS), and alkaline stress (15 mmol/L Na_2_CO_3_, AS). Leaves and roots were sampled at 24 h and 72 h for qRT‒PCR analysis of *OsRbohD* expression. Additionally, we aimed to clarify the expression pattern of *OsRbohD* under different stresses and its specificity in the SA stress response; thus, multiple treatments were included in the expression pattern analysis. Therefore, in the present study, rice seedlings at approximately the three-leaf stage were exposed to cold stress (4 °C, cold), heat stress (40 °C, heat), salt stress (120 mmol/L NaCl, saline), alkaline stress (15 mmol/L Na_2_CO_3_, alkaline), oxidative stress (25 µmol/L paraquat, PQ), 1% proanthocyanidins (PCs), 10 µmol/L abscisic acid (ABA), 10 µmol/L ABA inhibitor (fluridone, FLU), 10 µmol/L salicylic acid (SA), and 10 µmol/L MeJA (MeJA). All these stress factors or plant hormones can help confirm whether *OsRbohD* is specifically responsive to saline‒alkaline stress. Leaves were sampled at 24 h and 72 h for qRT‒PCR analysis of *OsRbohD* expression. Different tissues and organs of WT plants were collected throughout the growth stage to analyze the tissue-specific expression of *OsRbohD* by qRT‒PCR.

### RNA Isolation and Quantitative Real-Time PCR (qRT–PCR)

Rice samples were snap frozen in liquid nitrogen and ground using a benchtop ball mill (Scientz-48; Ningbo Scientz Biotechnology Co., Ltd., Ningbo, China) at 50 Hz for 30 s. Total RNA was extracted using the TRIzol reagent (TaKaRa Bio, Tokyo, Japan), and first-strand cDNA was synthesized using M-MLV reverse transcriptase (Thermo Fisher Scientific, Waltham, MA, USA) according to the manufacturers’ protocols. Gene-specific primers were designed using Primer 5.0 software (Table S1). The housekeeping gene *β-actin* (GenBank ID: X15865.1) was used as an internal standard. PCR amplification was performed as previously described (Liu et al. 2022b, 2025) using qTOWER2.2 (Analytic Jena, Germany). The relative expression levels were calculated using the 2^−ΔΔ*CT*^ method (Livak and Schmittgen 2001).

### Determination of Physiological and Biochemical Indices

#### Measurement of Chlorophyll Content

Chlorophyll content was measured as described by Wellburn and Lichtenthaler (1984), with some modifications (Liu et al. 2025). Briefly, rice leaf samples (0.1 g) were extracted using a 10-mL mixture of ethanol (5 mL) and acetone (5 mL). The absorbance of the supernatant was measured at 645 and 663 nm using a spectrophotometer (UV-2700; Shimadzu, Kyoto, Japan). The chlorophyll content (mg/g) was calculated using the following formula: (20.29 × A_645_ + 8.05 × A_663_) V/(1000 × W), where V is the extraction volume (mL) and W is the sample fresh weight (g).

### Measurement of the MDA Content

MDA content was determined via the thiobarbituric acid (TBA) reaction according to Heath and Packer (1968). A 0.1 g fresh leaf sample was homogenized in 1 mL of 50 mM phosphate buffer (pH 7.8) using a bench-top ball mill (Scientz-48, Ningbo Scientz Biotechnology Co., Ltd., Ningbo, China) at 50 Hz for 30 s and then centrifuged at 12,000 × g for 15 min. Subsequently, a 400 µL aliquot of the supernatant was mixed with 1 mL of 0.5% TBA, heated in a boiling water bath for 20 min, cooled, and centrifuged. The absorbance of the supernatant was measured at 450 nm, 532 nm, and 600 nm using a spectrophotometer (UV-2700; Shimadzu, Kyoto, Japan). The MDA concentration was calculated using the following formula:

$$ {\mathrm{6}}.{\mathrm{45}} \times \left( {{\mathrm{A}}_{{{\mathrm{532}}}} - {\mathrm{A}}_{{{\mathrm{6}}00}} } \right) - 0.{\mathrm{56}} \times {\mathrm{A}}_{{{\mathrm{45}}0}} $$

### Measurement of Membrane Injury (MI)

Membrane injury (MI) is a common and reliable indicator of membrane integrity and can be evaluated by measuring relative electrolyte leakage (Zhang et al. 2017). Relative electrolyte leakage can reflect the degree of cell membrane damage (Wei et al. 2015). Leaf samples (1 g fresh weight) were washed with deionized water to remove surface electrolytes and immersed in 15 mL of deionized water in 50 mL conical tubes at 20 °C for 1 h. The initial electrical conductivity (R1) was measured using a DDS-12 conductivity meter (Lida Inc., Shanghai, China). The samples were then boiled for 40 min to kill the tissues and cooled to 20 °C, after which the final electrical conductivity (R2) was measured. The MI was evaluated using the formula MI (%) =R1/R2 × 100%.

### Measurement of Na^+^, K^+^ and Other Mineral Element Contents

According to the experimental methods as described by Liu et al. (2022b), dried rice samples of different lines were cut into 5–10 mm fragments and subjected to complete digestion with an HNO_3_ and HClO_4_ (v: v = 2:1) mixture. The digested solution was diluted to 100 mL, and flame emission spectrometry (FP6410, Shanghai Precision and Scientific Instrument Co., Ltd., Shanghai, China) was applied to determine Na^+^ and K^+^ levels in shoots. Contents of mineral elements (Fe, Mn and Zn) were quantified via ICP-OES in accordance with relevant provisions specified in National Standard of the People’s Republic of China (GB 5009.268–2016, Determination of Multi-elements in Food Safety National Standards). Approximately 0.1 g rice sample was weighed into a polytetrafluoroethylene (PTFE) digestion tank, pre-digested overnight with 5 mL HNO₃. After sealed assembly, samples were sequentially heated at 80 ℃ for 1–2 h, at 120 ℃ for 1–2 h and then at 160 ℃ for 4 h in an oven. Upon natural cooling, acid was evaporated to near dryness. The digestion residue together with tank or cap washings were transferred into a 25 mL volumetric flask and diluted to volume for subsequent ICP-OES analysis.

### Measurement of ROS Levels

The O_2_•^-^ content was measured by monitoring nitrite formation from hydroxylamine in the presence of O_2_•^-^ (Elstner and Heupel 1976). The absorbance of the aqueous solution was measured at 530 nm to quantify the O_2_•^-^ content. The H_2_O_2_ content was measured by monitoring the absorbance of the titanium‒peroxide complex at 415 nm, as previously described by Brennan and Frenkel (1977). The absorbance values in the aqueous solution were measured at 415 nm to quantify the H_2_O_2_ content.

In accordance with the aforementioned theory and methods, the analytical reagents used to measure the H_2_O_2_ and O_2_•^-^ contents were acquired from a determination kit (Suzhou Michy Biomedical Technology Co., Ltd., China) following the manufacturer’s instructions (Liu et al. 2022b, 2025).

### Measurement of Plant Growth, Yield Components and Grain Yield at the Mature Stage

At the mature stage, the rice plants of different lines were harvested to determine or calculate the following parameters according to experimental methods described by Liu et al. (2023b, 2025): shoot length (SL), shoot dry weight (SDW), primary branches (PB) and secondary branches (SB) in panicles, panicle length (PL), panicle weight (PW), mean root length, root dry weight, and yield components, including panicle number (PN), spikelets per panicle (SP), number and weight of filled spikelets (FS) and empty spikelets (ES), percentage of filled spikelets (PFS), thousand-grain weight (TGW), harvest index (HI) and grain yield (GY). TGW and aboveground biomass were adjusted to a moisture content of 0.14 g/g (dry weight basis).

### Analysis of Expression Levels of Other *OsRbohs* Family Genes and Stress-Related Genes by qRT–PCR

Rice sample collection, RNA isolation, and first-strand cDNA synthesis were performed according to previously described methods. qRT–PCR was performed to determine the transcriptional levels of *OsRbohD* (Liu et al. 2012; Yoshiaki et al. 2005), *OsBI-1* (a cell death inhibitor gene) (Zhang et al. 2017), *OsKOD-1* (an inducer of programmed cell death) (Zhang et al. 2017; Liu et al. 2019), and six stress-related genes, namely, *OsHKT1* (a gene that codes for a Na^+^ transporter) (Kader et al. 2006), *OsHKT7* (a gene that codes for a K^+^ transporter) (Garciadeblás et al. 2003), *OsP5CS1* and *OsP5CS2* (which are involved in proline biosynthesis) (Junghe et al. 2004; Siriporn et al. 2013), *OsPEX11* (a gene that modulates the Na^+^/K^+^ transporter and reduces ROS accumulation in rice under salt stress) and *OsJRL* (a gene that plays important roles in cell protection and stress signal transduction) (Liu et al. 2022b). Furthermore, the expression levels of other *OsRbohs* family genes (*OsRbohA-OsRbohI*) (Yoshiaki et al. 2005; Wong et al. 2007; Jian et al. 2012; Yamauchi et al. 2017) in the different rice lines were measured by qRT–PCR. Gene-specific primers were designed using Primer 5.0 software (Table S1). The relative expression levels were calculated using the 2^−ΔΔ*CT*^ method (Livak and Schmittgen 2001).

### Experimental Design and Statistical Analyses

All the experiments were performed with five biological replicates. Statistical analyses were performed using the statistical software SPSS version 21.0 (IBM Corp., Armonk, NY, USA). One-way analysis of variance (ANOVA) and Duncan’s multiple range test were used to analyze the differences among the different lines, and *P* < 0.05 was considered to indicate statistical significance.

## Results

### Treatment with Rboh Inhibitors Reduced ROS Accumulation and ROS-Induced Damage

**Fig. 1:**
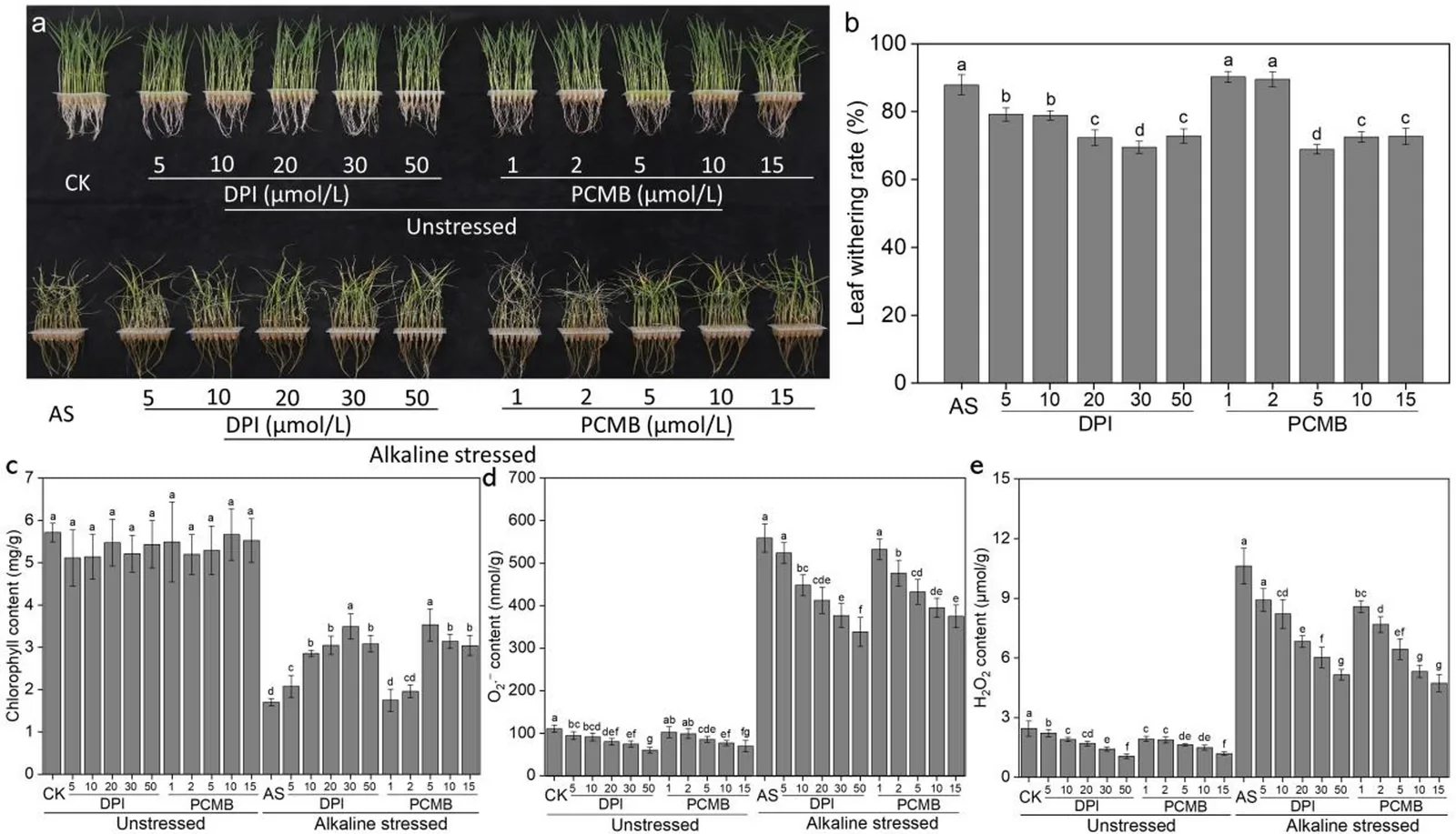
The application of diphenylene iodonium (DPI) or p-chloromercuribenzoate (PCMB) reduced ROS overaccumulation and improved alkaline stress in rice. Rice seedlings (cv. Nipponbare) at approximately the three-leaf stage were treated with deionized water (CK), alkaline stress (15 mmol/L Na_2_CO_3_), different concentrations of DPI (5, 10, 20, 30, 50 µmol/L) under unstressed conditions or alkaline-stressed conditions, and different concentrations of PCMB (1, 2, 5 10, 15 µmol/L) under unstressed conditions or alkaline-stressed conditions. **a** Photographs of the growth status of the rice seedlings were taken on the 7th day of the different treatments. **b** The leaf withering rate, **c** chlorophyll content, **d** O_2_•^-^ content and **e** H_2_O_2_ content were measured on the 7th day of the different treatments. The data are expressed as the mean ± SD; *n* = 5. Different letters on the columns represent significant differences (*P < 0.05*) between different treatments based on Duncan’s test

Exogenous diphenylene iodonium (DPI) or p-chloromercuribenzoate (PCMB) effectively reduced ROS overaccumulation in rice seedlings under alkaline stress, as evidenced by decreased O_2_•^-^ and H_2_O_2_ contents (Fig. 1). Compared with the alkaline stress (AS) treatment alone, these inhibitors enhanced the tolerance of rice to alkaline stress, characterized by a lower leaf withering rate and higher chlorophyll content. Under non-stress conditions, no significant differences in seedling growth were observed between the DPI/PCMB treatments (at all tested concentrations) and the control (CK) (Fig. 1a). Under alkaline stress, the leaf withering rate was reduced by 9.92–20.95% in the DPI treatment (5–50 µmol) and by 17.27–21.58% in the PCMB treatment (5–15 µmol/L) compared with the AS treatment (Fig. 1b). The chlorophyll content increased by 22.17–105.29% (DPI) and 78.94–107.59% (PCMB) under alkaline stress (Fig. 1c). Additionally, DPI and PCMB significantly decreased the O_2_•^-^ content by 14.93–45.68% and 7.80–37.39%, respectively, under non-stressed conditions and by 6.36–39.52% and 4.78–32.94%, respectively, under alkaline stress (Fig. 1d). Similarly, the H_2_O_2_ content decreased by 9.45–56.92% (DPI) and 21.30–51.36% (PCMB) under non-stressed conditions and by 16.04–51.41% (DPI) and 19.21–55.47% (PCMB) under alkaline stress (Fig. 1e). Under treatments with high concentrations of DPI and PCMB, the transcript levels of *OsRbohD* exhibited a mild reduction, with no statistically significant difference (Fig. S3a).

### Expression Analysis of *OsRbohD*

**Fig. 2:**
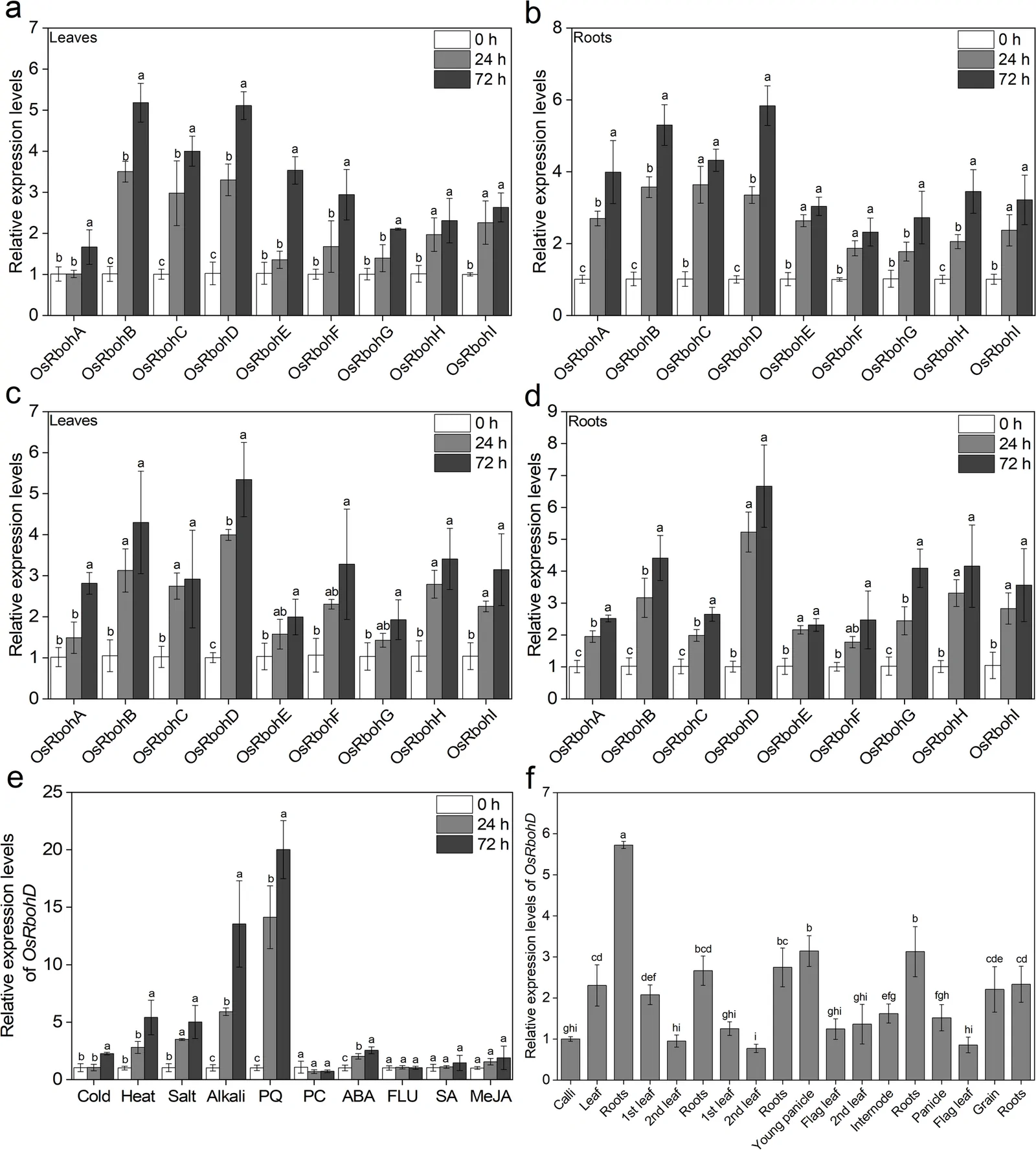
Gene expression pattern of *OsRbohD.* Rice seedlings at approximately the three-leaf stage were treated with deionized water (CK), saline stress (120 mmol/L NaCl) or alkaline stress (15 mmol/L Na_2_CO_3_, AS). The expression levels of the *OsRboh* family genes in leaves (**a**) or roots (**b**) under saline stress and in leaves (**c**) or roots (**d**) under alkaline stress were measured at 24 h and 72 h by qRT‒PCR. Rice seedlings at approximately the three-leaf stage were subjected to cold stress (4 °C), heat stress (40 °C), salt stress (120 mmol/L NaCl), alkali stress (15 mmol/L Na_2_CO_3_), oxidative stress (25 µmol/L paraquat, PQ), 1% proanthocyanidins (PC), 10 µmol/L absicsic acid (ABA), 10 µmol/L ABA inhibitor (fluridone, FLU), 10 µmol/L salicylic acid (SA), and 10 µmol/L MeJA. **e** Gene expression levels of *OsRbohD* in rice leaves under the above treatments were measured at 24 h and 72 h by qRT‒PCR. The expression levels at 0 h were set as the unit to calculate the expression levels. **f** The gene expression levels of *OsRbohD* in different tissues and organs of rice were measured by qRT‒PCR. The expression levels of the calli were set as the unit to calculate the expression levels. The data are expressed as the mean ± SD; *n* = 3. Different letters on the columns represent significant differences (*P* < 0.05) between different treatments based on Duncan’s test

Saline and alkaline stress upregulated the expression of all nine *OsRboh* family genes, with distinct tissue-specific expression patterns in leaves and roots. Among these, *OsRbohD* exhibited the highest induction under alkaline stress and a higher induction under saline stress, with expression levels increased by 37.237% and 431.96% in leaves at 72 h of saline and alkaline stress, respectively, and by 481.72% and 559.48% in roots at 72 h of saline and alkaline stress, respectively (Fig. 2a–d). *OsRbohD* was also strongly induced by multiple environmental stressors, including cold, heat, salt, alkali, and paraquat (PQ), with greater induction under PQ and alkaline treatments (Fig. 2e), whereas exogenous PCs suppressed *OsRbohD* expression. The application of ABA significantly increased *OsRbohD* expression, whereas the FLU, SA, and MeJA treatments had no statistically significant effects. Furthermore, *OsRbohD* was widely expressed across various rice tissues and organs, with particularly high expression levels in roots, leaves, and young panicles (Fig. 2f).

### Phenotypic Analysis of the *OsRbohD*-KO and *OsRbohD*-OE Lines

CRISPR/Cas9-mediated knockout of *OsRbohD* resulted in base deletions or insertions in the gene sequence. Specifically, the *OsRbohD*-KO-15 line harbored a 68-base deletion in the exon region of the *OsRbohD* open reading frame (ORF), whereas the *OsRbohD*-KO-7 line contained three insertion events (1 base, 82 bases, and 1 base) within the ORF (Fig. S2c). *OsRbohD* expression was significantly upregulated in the *OsRbohD*-overexpressing (*OsRbohD*-OE) lines compared with the wild-type (WT) plants (Fig. S3b). The expression levels of *OsRbohD* in the *OsRbohD*-KO lines were extremely low and did not significantly differ from those in the WT plants (Fig. S3b). Phenotypically, *OsRbohD*-KO lines exhibited slightly shorter plant heights, while *OsRbohD*-OE lines were marginally taller than WT plants (Fig. S4). Furthermore, apart from *OsRbohD*, the expression profiles of other *OsRbohs* family genes exhibited no significant differences among *OsRbohD*-KO, WT and *OsRbohD*-OE lines (Fig. S3c, d, e), indicating that the phenotypic variations observed in the tested lines are specifically modulated by *OsRbohD*.

### *OsRbohD*-KO Enhanced Seed Germination Under Saline and Alkaline Stress

**Fig. 3:**
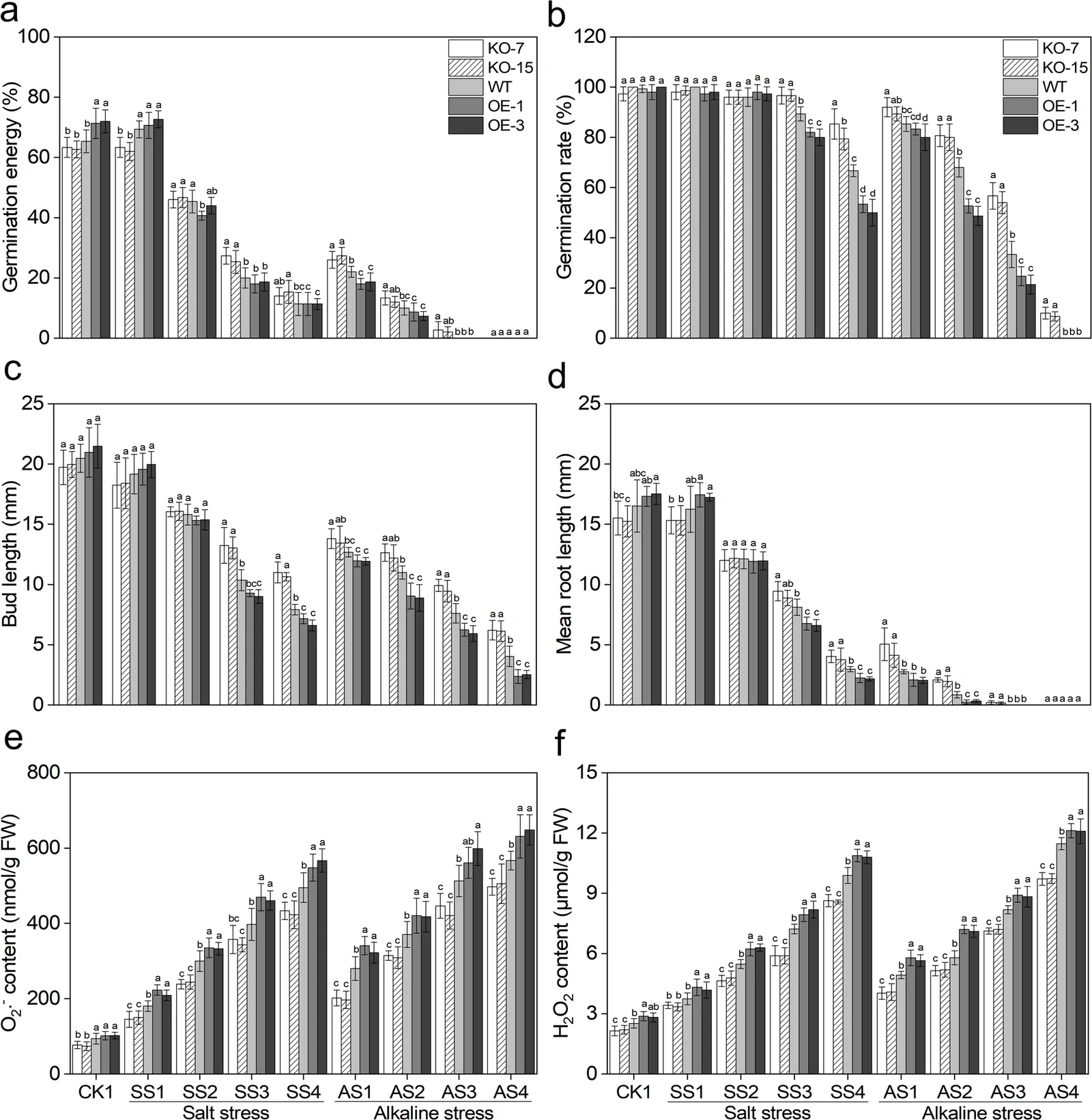
Germination status of the *OsRbohD*-KO, WT and *OsRbohD*-OE plants under salt and alkaline stress conditions. **a** The seed germination energy of the *OsRbohD*-KO, WT and *OsRbohD*-OE plants under normal (CK1), saline (SS1–SS4) and alkaline (AS1–AS4) stress was measured on the 3rd day. **b** The seed germination rates of the *OsRbohD*-KO, WT and *OsRbohD*-OE plants were measured on the 7th day under normal, salt and alkaline stress conditions. **c** Bud length, **d** mean root length, **e** O_2_•^-^ content and **f** H_2_O_2_ content of the *OsRbohD*-KO, WT and *OsRbohD*-OE plants were measured on the 7th day under normal, salt and alkaline stress conditions. The data are expressed as the mean ± SD; *n* = 5. Different letters on the columns represent significant differences (*P < 0.05*) between different lines of the same treatment based on Duncan’s test.

Under the CK and SS1 treatments, *OsRbohD*-KO inhibited seed germination, as indicated by lower germination energy, shorter bud length, and reduced mean root length compared to WT plants, whereas *OsRbohD*-OE promoted germination (Fig. 3a, c, d, S5). No significant differences in the germination rate were observed among all the lines under the CK, SS1, and SS2 treatments on the 7th day of germination (Fig. 3b). However, under the SS3, SS4, and all -alkaline stress (AS1-AS4) treatments, the *OsRbohD*-KO lines presented significantly higher germination rates. Specifically, the germination rates were increased by 8.21% (SS3), 19.00–28.0% (SS4), and 4.69-70.00% (AS1-AS3) in the *OsRbohD*-KO lines (Fig. 3b) compared with the WT plants. *OsRbohD*-KO also significantly increased bud length by 1.52–38.89% under salt stress (SS2-SS4) treatments and by 5.99–53.47% under alkaline stress (AS1-AS4) treatments (Fig. 3c), and it increased root length by 9.36–36.49% under salt stress (SS3-SS4) treatments and 49.28-147.62% under alkaline stress treatments (AS1-AS2) (Fig. 3d) compared with the WT plants.

Consistently, the *OsRbohD*-KO lines accumulated significantly lower levels of O_2_•^-^ and H_2_O_2_ than the WT plants. The O_2_•^-^ content was reduced by 10.04–20.38% (saline stress) and 10.89–29.77% (alkaline stress) (Fig. 3e), while H_2_O_2_ content was decreased by 8.66–18.47% (saline stress) and 10.29–18.27% (alkaline stress) in the *OsRbohD*-KO lines (Fig. 3f). In contrast, the *OsRbohD*-OE lines accumulated higher levels of O_2_•^-^ and H_2_O_2_ under all the stress treatments.

### *OsRbohD*-KO Alleviated Leaf Withering and Oxidative Damage in Rice Seedlings Under Saline and Alkaline Stress

**Fig. 4:**
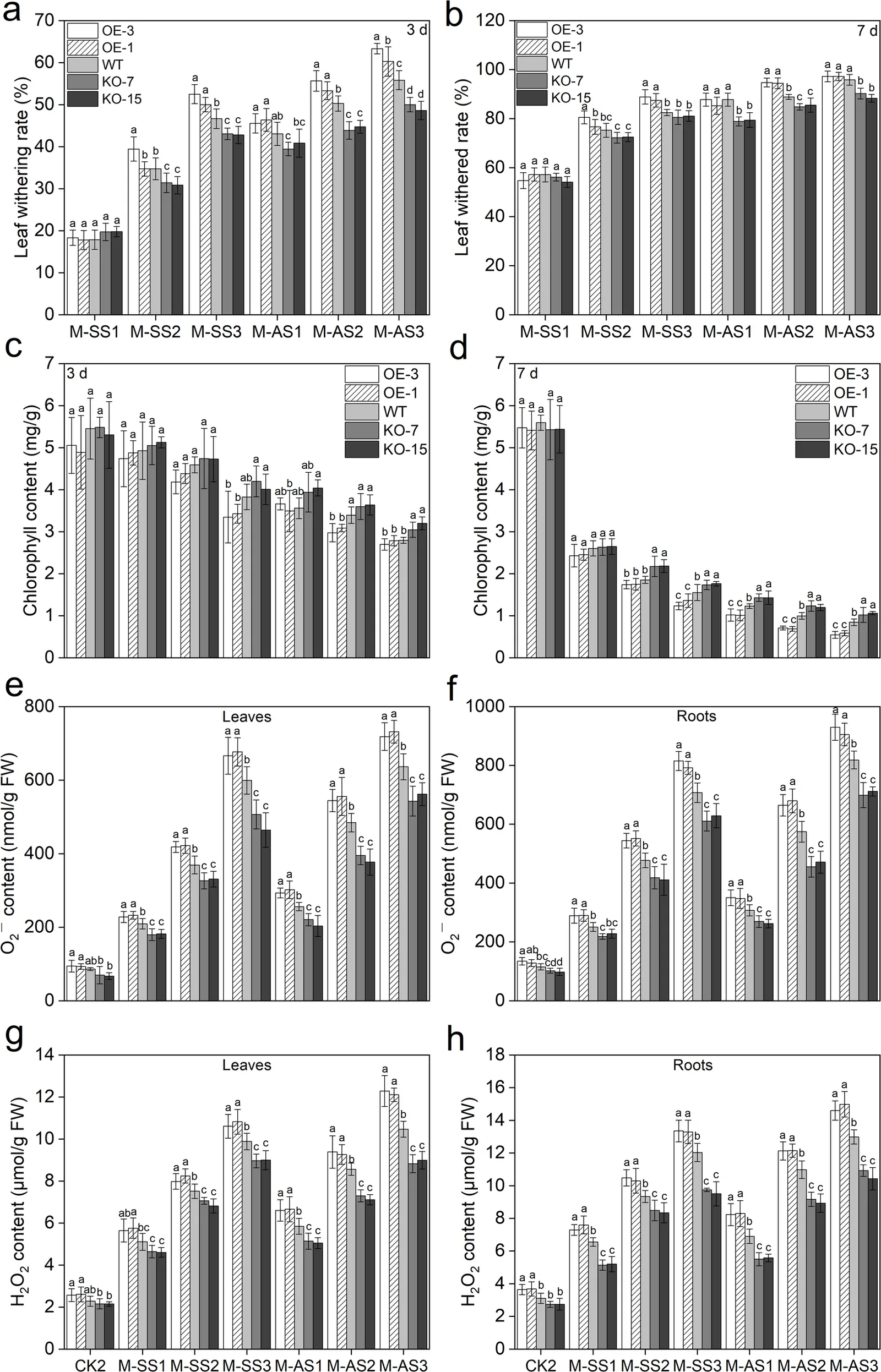
*OsRbohD*-KO alleviated leaf withering in rice seedlings under saline and alkaline stress conditions. Rice seedlings of different lines at approximately the three-leaf stage were treated with distilled water (CK2), saline stress stimulated by 50, 100, or 150 mmol/L NaCl (M-SS1, M-SS2, M-SS3), and alkaline stress stimulated by 10, 15, or 20 mmol/L Na_2_CO_3_ (M-AS1, M-AS2, M-AS3, respectively). **a** Leaf withering rates were measured on the 3rd day (**a**) and on the 7th day (**b**) of different treatments. **c** Chlorophyll content in leaves was measured on the 3rd day (**c**) and on the 7th day (**d**) of different treatments. The O_2_•^-^ content in leaves (**e**) and roots (**f**) was measured on the 7th day of the different treatments. The H_2_O_2_ contents in leaves (**g**) and roots (**h**) were measured on the 7th day of the different treatments. The data are expressed as the mean ± SD; *n* = 5. Different letters on the columns represent significant differences (*P < 0.05*) between different lines of the same treatment based on Duncan’s test.

*OsRbohD*-KO significantly alleviated leaf withering in rice seedlings under salt and alkaline stress, as reflected by lower leaf withering rates compared to WT plants (Fig. S6, S7, S8). Compared with those in the WT plants, the leaf withering rates in the *OsRbohD*-KO lines decreased by 7.74–11.20% and 5.16–12.94% on the 3rd day of saline stress (M-SS1 to M-SS3 treatments) and alkaline stress (M-AS1 to M-AS3 treatments), respectively (Fig. 4a). The reduction was 1.68–5.34% (saline stress) and 3.75–10.13% (alkaline stress) on the 7th day (Fig. 4b). Furthermore, *OsRbohD*-KO significantly increased the chlorophyll content by 2.51–9.66% (saline stress) and 6.04–14.41% (alkaline stress) on the 3rd day and by 1.09–17.58% (saline stress) and 16.41–25.59% (alkaline stress) on the 7th day (Fig. 4c, d) relative to the WT plants. Conversely, the *OsRbohD*-OE lines presented higher leaf withering rates and lower chlorophyll contents under salt and alkaline stress.

The O_2_•^-^ content was reduced by 9.11–22.54% under saline stress and by 11.70–22.12% under alkaline stress in the *OsRbohD*-KO lines (Fig. 4e, f) compared with the WT plants. Consistently, the H_2_O_2_ content was reduced by 6.06–21.65% under saline stress and by 12.08–20.24% under alkaline stress in the *OsRbohD*-KO lines (Fig. 4g, h) compared with the WT plants. Furthermore, *OsRbohD*-KO mitigated membrane injury, as shown by lower malondialdehyde (MDA) content and membrane injury (MI). Compared with that in the WT plants, the MDA content in the *OsRbohD*-KO lines was reduced by 11.56–18.02% under saline or alkaline stress conditions (Fig. S8a), and it decreased by 2.28–10.40%, 8.39%, 6.05%, 8.43% and 7.17% under saline or alkaline stress conditions (Fig. S8b). In contrast, the *OsRbohD*-OE lines accumulated higher MDA content and exhibited higher MI under saline and alkaline stress.

However, re-watering after 6 days of saline-alkali stress treatments failed to alleviate seedling withering. *OsRbohD*-KO lines exhibited better growth status than WT and *OsRbohD*-OE lines (Fig. S9). The leaf withering rates and chlorophyll content in the *OsRbohD*-KO lines were decreased by 2.63–10.33% and 6.30–20.51%, compared to WT plants, whereas those of the *OsRbohD*-OE lines showed a 1.41–3.00% and 11.90–18.54% reduction (Fig. S9, S10).

### *OsRbohD*-KO Alleviated Ionic Toxicity and Promoted the Uptake of Mineral Elements

As shown in Fig. S11, *OsRbohD*-KO alleviated ionic toxicity in rice seedlings under saline and alkaline stress conditions as shown by the lower Na^+^ contents, Na^+^/K^+^ ratio, and higher K^+^ contents. Compared with the WT plants, the Na^+^ contents of the *OsRbohD*-KO lines were decreased by 3.40–8.88% or 11.03–22.13% under saline or alkaline stress conditions (Fig. S11a); the K^+^ contents were increased by 2.51–5.45% or 2.50–20.10% under saline or alkaline stress conditions (Fig. S11b); whereas the *OsRbohD*-OE lines presented increases of Na^+^ contents and decreases of K^+^ contents. Consistently, *OsRbohD*-KO promoted the uptake of mineral nutrients in rice under SA stress conditions. Contents of Fe, Mn and Zn were significantly higher in *OsRbohD*-KO lines than those in the WT plants and *OsRbohD*-OE lines (Fig. S11d–f). Notably, the increments in these three mineral elements in *OsRbohD*-KO seedlings were substantially greater under alkali-specific stress.

### *OsRbohD*-KO Improved Seedling Growth in Saline-Alkaline Soils

**Fig. 5:**
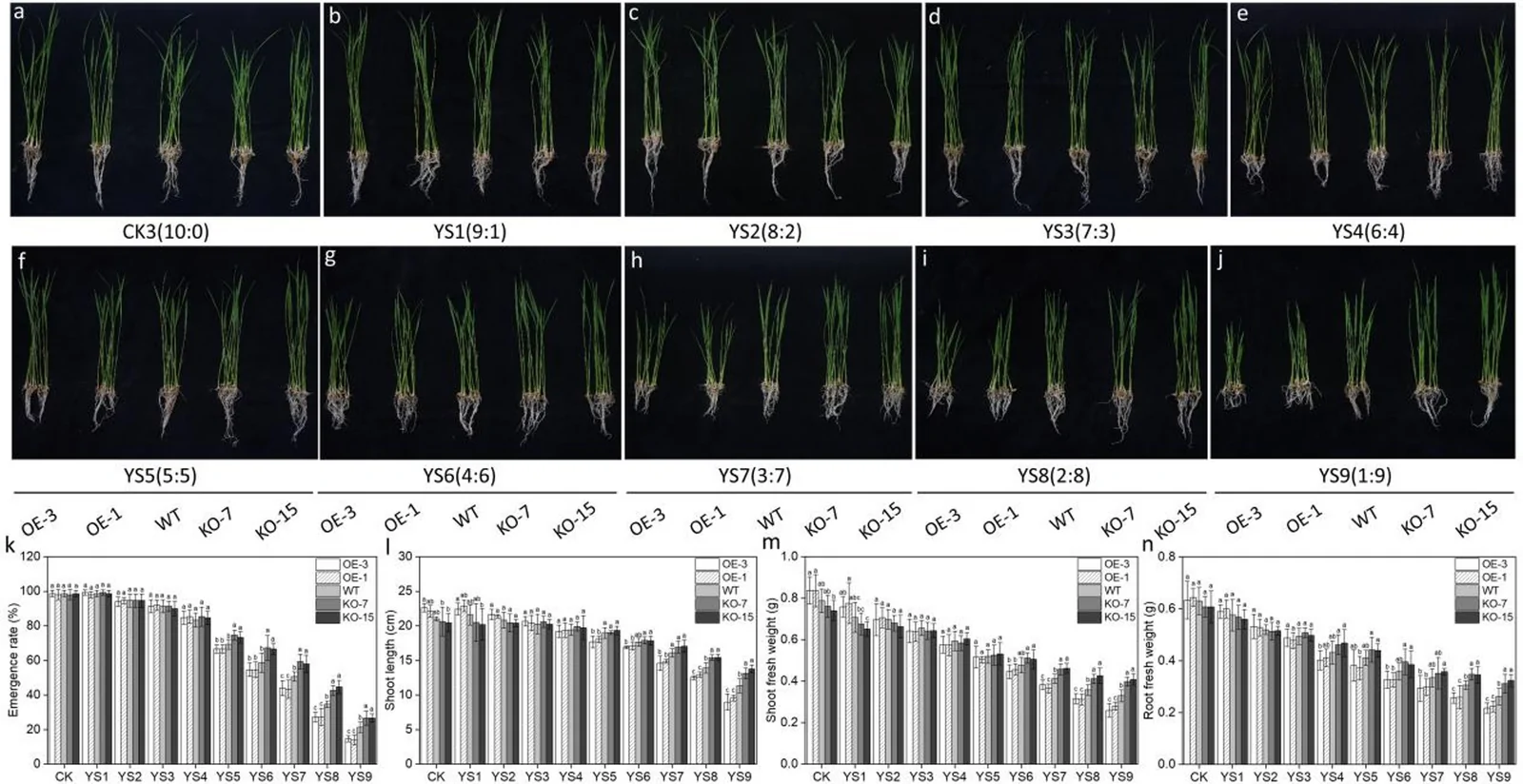
*OsRbohD*-KO improved the growth of rice seedlings in saline-alkaline soils. Germinated rice seeds of different lines were sown in seedling pots containing saline-alkaline soils to different degrees. The pH values of the different saline-alkaline soils were 7.53 (CK3), 8.11 (YS1), 8.52 (YS2), 8.84 (YS3), 9.10 (YS4), 9.33 (YS5), 9.49 (YS6), 9.78 (YS7), 10.01 (YS8), and 10.27 (YS9). Photographs of the growth status of the rice seedlings were taken on the 10th day of the different treatments (**a–j**). **k** Emergence rate, **l** shoot length, **m** shoot fresh weight, and **n** root fresh weight were measured on the 10th day of the different treatments. The data are expressed as the mean ± SD; *n* = 5. Different letters on the columns represent significant differences (*P < 0.05*) between different lines of the same treatment based on Duncan’s test.

As shown in Fig. 5, under the CK, YS1, YS2 and YS3 treatments, *OsRbohD*-OE improved seedling growth, as shown by the greater shoot length, shoot weight and root weight in the *OsRbohD*-OE lines. However, under severe SA soil (YS5-YS9 treatments), the growth performance of the *OsRbohD*-KO lines was superior, with increased seedling vigor (Fig. 5a–j, S12), emergence rate (Fig. 5k), shoot length (Fig. 5l), shoot fresh weight (Fig. 5m), and root fresh weight (Fig. 5n). Compared with that of the WT plants, the emergence rate of the *OsRbohD*-KO lines increased by 5.77–28.85% under the YS5-YS9 treatments, whereas that of the *OsRbohD*-OE lines showed a 3.85–34.38% reduction (Fig. 5k). Compared with those of the WT plants, the shoot length, shoot fresh weight, and root fresh weight of the *OsRbohD*-KO lines were increased by 0.40–21.58%, 1.15–23.64% and 6.80–23.66%, respectively, under the YS5-YS9 treatments, whereas those of the *OsRbohD*-OE lines showed reductions of 4.79–20.80%, 0.77–21.21% and 7.28–16.79%, respectively (Fig. 5l, m, n).

### *OsRbohD*-KO Enhanced Grain Yield and Root Growth in Saline-Alkaline Soils

Under the CK and SP1 treatments, no significant differences in plant growth were observed among the *OsRbohD*-KO, WT, and *OsRbohD*-OE lines at the tillering, heading, and filling stages (Fig. S13–S15). However, under the SP2 and SP3 treatments, *OsRbohD*-KO improved plants’ growth, with greater plant length occurring during the growth stage (Fig. S13–S15).

**Fig. 6:**
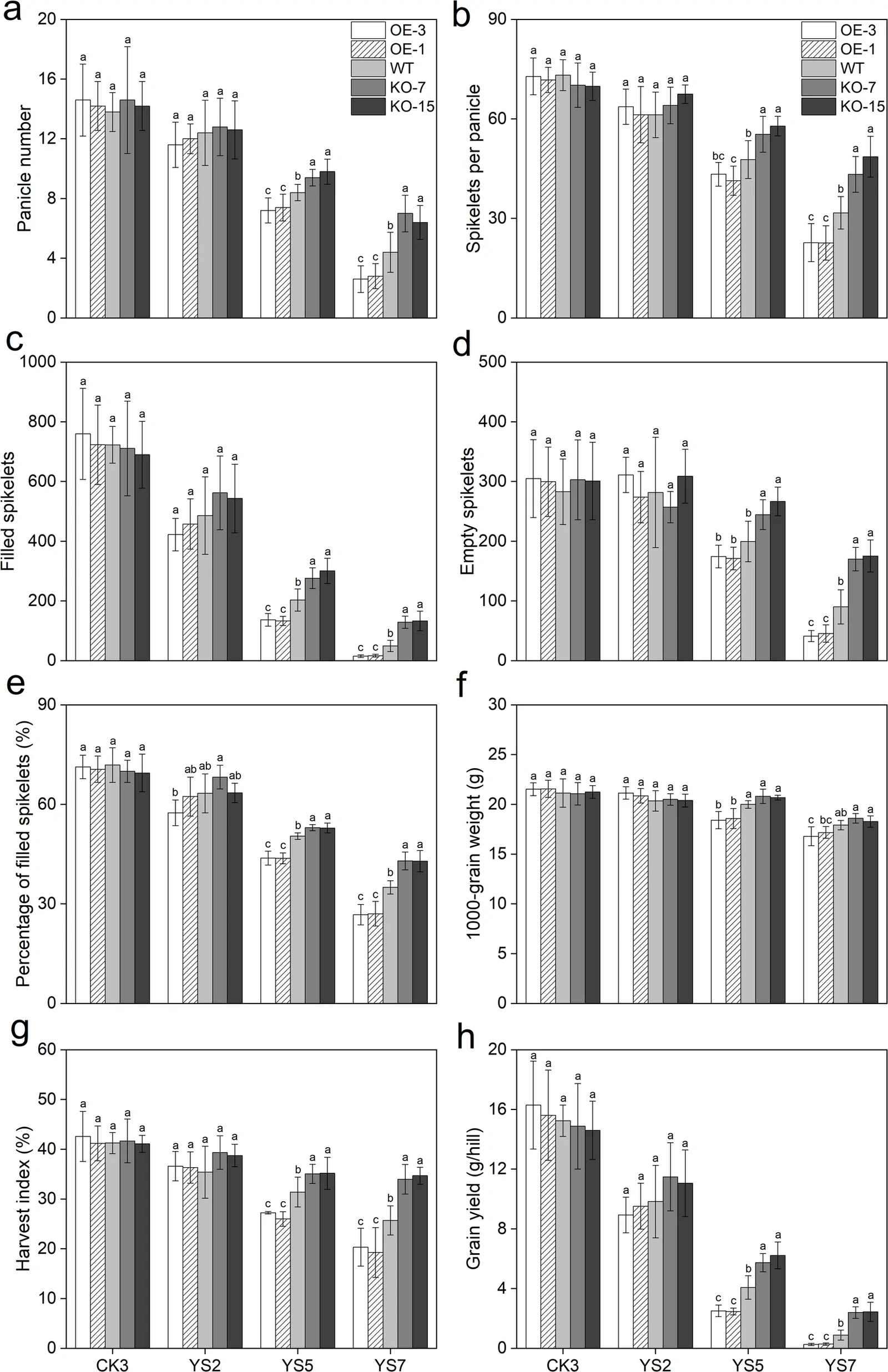
Yield components and grain yield of *OsRbohD*-KO, WT and *OsRbohD*-OE plants at the mature stage under different treatments. CK3: normal soil with a pH of 7.53; YS2: saline-alkaline soil with a pH of 8.52; YS5: saline-alkaline soil with a pH of 9.33; and YS7: saline-alkaline soil with a pH of 9.78. **a** Panicle number (PN), **b** spikelets per panicle (SP), **c** filled spikelets (FS), **d** empty spikelets (ES), **e** percentage of filled spikelets (PFS), **f** 1000-grain weight (TGW), **g** harvest index (HI) and **h** grain yield (GY) were measured at the mature stage. The data are expressed as the mean ± SD; *n* = 5. Different letters on the columns represent significant differences (*P < 0.05*) between different lines of the same treatment based on Duncan’s test.

At the mature stage, no significant differences in growth parameters or yield components were detected among the lines under the CK and SP1 treatments (Fig. 6, S16-S18). In the SP2 and SP3 treatments, *OsRbohD*-KO significantly enhanced plant growth and yield-related traits. Compared with those in the WT plants, the shoot dry weight, primary branches, secondary branches, panicle length, and panicle weight in the *OsRbohD*-KO line were increased by 17.38–54.20%, 9.09–51.93%, 7.74–119.35%, 3.21–15.59% and 16.94–48.62%, respectively, while the *OsRbohD*-OE lines reduced these indexes by 9.10-46.95%, 7.14–33.47%, 9.68–44.89%, 3.30-18.05% and 17.88–49.31% (Fig. S18). *OsRbohD*-KO also increased yield components under the SP2 and SP3 treatments, including panicle number (11.90–59.09%), number of spikelets per panicle (16.02–53.29%), number of filled spikelets (35.79–170.04%), percentage of filled spikelets (4.85–22.76%), and 1000-grain weight (2.02–4.10%), compared with the WT plants (Fig. 6). However, the *OsRbohD*-OE lines presented decreases of 10.91–11.90%, 9.25–28.69%, 35.79–170.04%, 13.17–23.52% and 4.12–7.93% in the above indices (Fig. 6). Additionally, compared with the WT plants, the *OsRbohD*-KO increased the harvest index and grain yield by 13.26–25.07% and 38.34–71.17%, respectively, under the SP2 and SP3 treatments, whereas the *OsRbohD*-OE lines presented decreases of 11.61–34.96% and 40.79–175.23%, respectively (Fig. 6).

*OsRbohD*-KO also improved root growth under severe SA stress (Fig. S19). Compared with the WT plants, the *OsRbohD*-KO lines presented 9.39–38.62% longer root length and 30.69–52.72% greater root weight under the SP2 and SP3 treatments, whereas the *OsRbohD*-OE lines showed reductions of 2.52–15.86% (root length) and 20.05–52.59% (root weight) (Fig. S19).

### *OsRbohD*-KO Upregulated Stress-Related Genes Under Severe Saline-Alkaline Stress

**Fig. 7:**
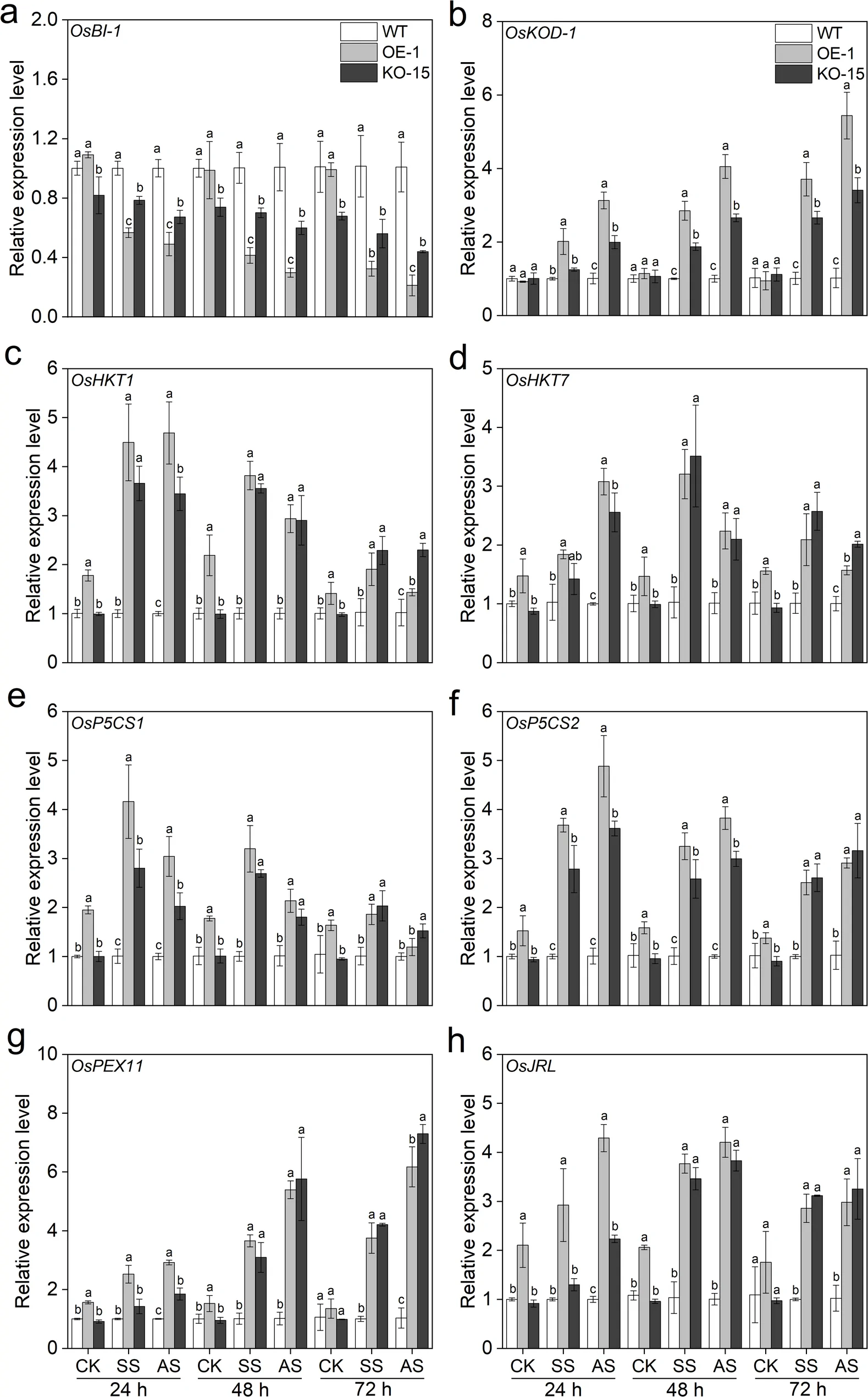
Relative gene expression levels of stress-related genes in *OsRbohD*-KO (KO-15), WT and *OsRbohD*-OE (OE-1) rice seedlings. Rice seedlings of different lines at approximately the three-leaf stage were treated with distilled water (CK), saline stress stimulated by 100 mmol/L NaCl (SS), and alkaline stress stimulated by 15 mmol/L Na_2_CO_3_ (AS). Gene expression levels of the stress-related genes *OsBI-1* (**a**), *OsKOD-1* (**b**), *OsHKT1* (**c**), *OsHKT7* (**d**), *OsP5CS1* (**e**), *OsP5CS2* (**f**), *OsPEX11* (**g**), and *OsJRL* (**h**) in rice leaves were measured at 24 h, 48 h and 72 h of the above treatments by qRT‒PCR. The expression levels in WT plants were set as the unit to calculate the expression levels under each treatment. The data are expressed as the mean ± SD; *n* = 3. Different letters on the columns represent significant differences (*P* < 0.05) between different lines based on Duncan’s test.

*OsRbohD*-OE induced the expression of stress-related genes under CK conditions or during the early stage of saline or alkaline stress, but this induction was suppressed with prolonged stress exposure (Fig. 7). The expression of the cell death inhibitor gene *OsBI-1* was downregulated by saline or alkaline stress, but its expression level remained higher in the *OsRbohD*-KO lines than in the *OsRbohD*-OE lines (Fig. 7a). In contrast, the expression of the cell death-promoting gene *OsKOD-1* was induced by saline or alkaline stress, with a more pronounced induction in the *OsRbohD*-OE lines (Fig. 7b). With prolonged stress (72 h), the expression levels of stress-responsive genes, such as *OsHKT1*, *OsHKT7*, *OsP5CS1*, *OsP5CS2*, *OsPEX11*, and *OsJRL*, were significantly increased in the *OsRbohD-*KO lines. Compared with those in the WT plants, the expression levels of *OsHKT1*, *OsHKT7*, *OsP5CS1*, *OsP5CS2*, *OsPEX11*, and *OsJRL* in the *OsRbohD*-KO lines were increased by 122.90%, 155.02%, 101.26%, 160.55%, 319.81%, and 211.59%, respectively, at 72 h of saline stress and by 124.99%, 100.39%, 52.09%, 207.94%, 624.71%, and 218.06%, respectively, at 72 h of alkaline stress. In contrast, the *OsRbohD*-OE lines exhibited lower induction rates—85.70%, 107.09%, 84.19%, 150.94%, 274.52%, and 185.73% at 72 h of saline stress and 40.39%, 56.23%, 19.03%, 183.35%, 496.10%, and 191.31% at 72 h of alkaline stress (Fig. 7c–h).

## Discussion

Saline-alkaline (SA) soil has caused widespread ecological degradation and substantial crop yield losses globally, with adverse effects primarily attributed to SA stress (Lv et al. 2013; Wei et al. 2015). As a complex abiotic stress, SA stress inhibits rice growth through diverse mechanistic pathways, with ROS overaccumulation in plant cells recognized as one of the most detrimental factors (Feng et al. 2024; Xu et al. 2025). Previous studies have linked SA stress-induced root damage in rice to ROS overaccumulation (Zhang et al. 2017; Liu et al. 2019), which is associated with the upregulation of ROS production-related genes, and these findings have been validated in the response of rice to other environmental stresses (Liu et al. 2023b, 2025). The exogenous application of antioxidants has been shown to alleviate root damage and leaf withering in rice under SA stress by reducing ROS levels (Zhang et al. 2017). These studies collectively confirm that reducing ROS accumulation under alkaline stress is an important and effective strategy for enhancing the tolerance of rice to SA stress (Liu et al. 2019, 2022b). In the present study, exogenous application of Rboh inhibitors (DPI and PCMB) significantly decreased the ROS levels in rice seedlings under alkaline stress (Fig. 1) and alleviated seedling wilting (Fig. 1), indicating that DPI and PCMB inhibited the activity of NADPH oxidase and thereby reduced ROS accumulation, which was consistent with previous studies (Gestelen et al. 1997; Yoshioka et al. 2001). These results confirmed the vital role of Rboh in the response of rice to SA stress, such as alkaline stress stimulated by ≥ 15 mmol/L Na_2_CO_3_. These results are consistent with previous studies which showed that Rboh genes may play a negative regulatory role in rice response to multiple severe environmental stress factors, including heat, salinity, and alkalinity (Shen et al. 2023; Chen et al. 2024; Liu et al. 2025).

*OsRboh* family genes are the primary contributors to ROS production in rice (Wang et al. 2013; Liu et al. 2025) and play critical roles in regulating plant responses to various environmental stress factors, such as heat, drought, salinity and alkalinity (Shi et al. 2020; Chen et al. 2024; Liu et al. 2025). However, different members of the *OsRboh* gene family respond differently to diverse environmental stresses (Wang et al. 2016b; Liu et al. 2025). Our results revealed that SA stress induces the expression of *OsRboh* family genes, with different members showing distinct expression patterns (Fig. 2). Among the nine *OsRboh* family members, *OsRbohD* exhibited the highest expression level under saline and alkaline stress (Fig. 2), indicating that it is the dominant gene mediating SA stress-induced ROS production. Knockout or overexpression of a specific gene via CRISPR/Cas9 or transgenic technology can effectively improve SA tolerance in rice (Ndudzo et al. 2024; Guo et al. 2025). Consequently, we employed CRISPR/Cas9 technology to knock out *OsRbohD* for generating *OsRbohD*-knockout (*OsRbohD*-KO) lines, and generated *OsRbohD*-overexpression (*OsRbohD*-OE) lines by transgenic technology. Phenotypic and physiological analyses revealed that *OsRbohD*-KO significantly reduced ROS levels in seeds (Fig. 3), leaves (Fig. 4), and roots (Fig. 4). This reduction in ROS accumulation alleviated oxidative stress and improved SA stress tolerance, as evidenced by increased seed germination rates and root lengths (Fig. 3, S5), increased seedling survival rates and chlorophyll contents (Fig. 4, S6, 7), alleviated membrane injury (Fig. S8), and enhanced plant growth (Fig. 5, S12–S19) and grain yield (Fig. 6) in SA soils. The *OsRbohD*-OE lines exhibited high sensitivity to SA stress, especially under severe SA stress conditions. Additionally, *OsRbohD*-KO has a pronounced alleviating effect on alkalinity stress tolerance in rice seeds and seedlings. *OsRbohD*-KO significantly elevated seed germination rate and leaf survival rate across all tested alkaline concentrations, and this promotive effect was further intensified with increasing stress levels. These observations are also consistent with the aforementioned finding that *OsRbohD* displayed the highest transcript abundance under alkaline stress (Fig. 2). Consistently, no significant differences were observed in the expression patterns of other *OsRboh* family genes among *OsRbohD*-KO, and WT plants (Fig. S3), indicating that the loss of *OsRbohD* did not trigger compensatory upregulation of paralogs. Furthermore, *OsRbohD*-KO significantly upregulated the expression of various stress-responsive genes (Fig. 7), priming the gene regulatory network for stress adaptation. Collectively, these results demonstrate that CRISPR/Cas9-mediated knockout of *OsRbohD* enhances rice SA tolerance by reducing ROS levels under severe SA stress, providing valuable genetic resources for the molecular breeding of SA-tolerant rice varieties. Previous studies have revealed that the exogenous antioxidants improve rice SA tolerance and other *OsRboh* family gene members negatively regulated rice SA tolerance, and both effects are associated with alleviating excessive ROS accumulation (Zhang et al. 2017; Shen et al. 2023), which is consistent with our finding that *OsRbohD*-KO enhances SA tolerance by reducing ROS accumulation, which further supports the key role of ROS homeostasis in the response of rice to SA stress. In addition, the growth performance of rice seedlings of different lines failed to recover after re-watering (Fig. S9, S10), indicating that SA stress caused irreversible damage to rice seedlings. This phenomenon may be associated with the massive accumulation of toxic ions (Fig. S11) and ROS (Fig. 4) in seedling cells. *OsRbohD*-KO reduced Na^+^ accumulation and improved mineral nutrient uptake in rice seedlings under SA stress conditions (Fig. S11). These beneficial effects were likely attributable to maintained plasma membrane integrity (Fig. S8) and upregulated expression of ion transporter-related genes (Fig. 7), which is consistent with previous findings (Shen et al. 2023). Nevertheless, the *OsRbohD*-KO lines exhibited better growth status than the WT and *OsRbohD*-OE lines, which indicates that the knockout of *OsRbohD* can enhance the SA tolerance of rice to a certain extent. However, such a mitigation effect is insufficient under prolonged or extremely severe SA stress conditions. Therefore, more efficient strategies need to be explored in practical agricultural production to improve rice yield in SA soils.

Saline-alkaline (SA) soils are characterized by low productivity, with crops grown in such soils exhibiting various growth-suppressive phenotypes, including poor seedling emergence, slow growth, and severe yield loss (Fang et al. 2021). Rice grown in SA soils experiences significant reductions in seedling survival, tiller number, panicle number, and 1000-grain weight, resulting in yield losses exceeding 30% (Wei et al. 2017; Liu et al. 2022b). Our previous study revealed that residual sodium carbonate (Na_2_CO_3_) in SA soils during the reproductive stage was the primary limiting factor for rice yield, with yields reaching only 12.9–90.7% of the global average (Seck et al. 2012; Wang et al. 2018). In the present study, rice seedlings (Fig. 5) and mature plants (Fig. 6) grown in SA soils experienced severe growth inhibition and yield loss. However, *OsRbohD*-KO significantly improved seedling emergence and growth (Fig. 5), as well as plant growth throughout the entire growth cycle (Fig. S13–S17), yield components (Fig. 6, S18), and root growth (Fig. S19). These results confirm the growth-promoting and yield-enhancing effects of *OsRbohD*-KO in rice under SA stress. Notably, under control and moderate SA stress conditions, the *OsRbohD*-KO lines exhibited slightly weaker growth than the WT or *OsRbohD*-OE lines. However, as the severity of SA stress increased, the growth and yield of the *OsRbohD*-KO lines significantly improved, indicating that appropriate ROS levels are critical for regulating crop stress responses. Moderate ROS levels promote crop growth and development, while stress-induced excessive ROS accumulation causes severe cellular damage, metabolic disruption, and ultimately growth inhibition and yield loss, which is in accordance with previous studies (Liu et al. 2025).

Rice employs complex gene expression regulatory networks to cope with various stressors, including genes that are involved in antioxidant defense, cellular protection, ion transport, and the synthesis of osmoregulatory substances (Zhang et al. 2017; Liu et al. 2019). Different ROS levels have been shown to participate in the regulation of gene networks associated with stress responses in rice (Liu et al. 2025). In the present study, saline or alkaline stress significantly upregulated the expression of abiotic stress-related genes involved in osmotic stress adaptation (*OsP5CS1* and *OsP5CS2*), ionic homeostasis (*OsHKT1*, *OsHKT7* and *OsPEX11*), and cellular defense (*OsJRL*), with greater upregulation observed in the *OsRbohD*-OE lines during the early stage of stress exposure (before 24 h) (Fig. 7). However, as the duration of stress increased, the expression of these genes increased significantly in the *OsRbohD*-KO lines. These results indicate that appropriately increasing ROS levels during the early stage of SA stress promotes signal transduction in rice, which is involved in osmotic adjustment, ion transport and oxidative stress, thereby regulating rice growth. However, prolonged or severe SA stress can cause disorders in these metabolic processes through the induction of excessive ROS accumulation (Zhang et al. 2017; Liu et al. 2019). Consequently, the knockout of *OsRbohD* reduced ROS levels and modulated these metabolic processes to enable rice to adapt to SA stress. Additionally, the *OsRbohD*-KO lines exhibited lower expression of cell death-related genes (*OsKOD-1*) and higher expression of cell death-inhibiting genes (*OsBI-1*) (Fig. 7), indicating that lower ROS levels alleviate excessive cell death in rice under severe SA stress. Collectively, these data demonstrate that *OsRbohD*-KO confers resistance to severe SA stress by alleviating the oxidative damage caused by excessive ROS accumulation, which in turn maintains appropriate expression levels of abiotic stress-related genes and thereby improves the adaptive capacity of the rice plants to SA stress.

*RbohD* is involved in immune responses in various plants, including Arabidopsis, rice, and potato (Hu et al. 2020). *AtRbohD* and *NtRbohD* participated in the signal transduction of fungal infection and immune responses in *Arabidopsis thaliana* and potato by regulating ROS and phytohormone levels (Dubiella et al. 2013; Liu et al. 2015). In rice, fungi induced ROS production by mediating NADPH oxidase, which served as a key cause for the occurrence of rice blast disease, including *RbohD* (Egan et al. 2007). The ATP synthase *β* subunit ATPb interacts with *OsRbohD* and loss of ATPb function led to *OsRbohD*-dependent NADPH accumulation, which further triggers ROS bursts and participating in *Pix*-mediated resistance against *Magnaporthe oryzae* in rice (Xiao et al. 2024; Mondal et al. 2024). Collectively, these previous findings, including the results of our present study, further demonstrated the important role of *RbohD* in plants response to both biotic and abiotic stresses, which provided a solid theoretical foundation for the genetic improvement of rice stress resistance through gene editing strategies.

In summary, in this study, we showed that CRISPR/Cas9-mediated knockout of *OsRbohD* reduced the excessive accumulation of ROS in rice under severe SA stress conditions, alleviated oxidative damage, and enhanced rice tolerance to SA stress. It also promoted plant growth and grain yield in severe SA soils. These findings provide important gene targets for the molecular breeding design of SA-tolerant rice varieties.

## Supplementary Information

Below is the link to the electronic supplementary material.

Supplementary Material 1.

## Data Availability

All data generated or analyzed during this study are included in this published article and the supplementary information files.

## References

[CR1] Brennan T, Frenkel C (1977) Involvement of hydrogen peroxide in the regulation of senescence in pear. Plant Physiol 59(3):411–416. 10.1104/pp.59.3.411 16659863 PMC542414

[CR2] Chen Y, Zhang R, Wang R, Li J, Wu B, Zhang H, Xiao G (2024) Overexpression of OsRbohH enhances heat and drought tolerance through ROS homeostasis and ABA mediated pathways in rice (Oryza sativa L). Plants 13(17):2494. 10.3390/plants13172494 39273977 PMC11397177

[CR3] Choudhury FK, Rivero RM, Blumwald E, Mittler R (2017) Reactive oxygen species, abiotic stress and stress combination. Plant J 90(5):856–867. 10.1111/tpj.13299 27801967

[CR4] Dong J, Yu XH, Dong J et al (2024) An artificially evolved gene for herbicide-resistant rice breeding. Proc Natl Acad Sci U S A 121(134):e240728512111. 10.1073/pnas.2407285121 PMC11348328 39133859

[CR5] Dubiella U, Seybold H, Durian G, Komander E, Lassig R, Witte CP, Schulze WX, Romeis T (2013) Calcium-Dependent Protein Kinase/NADPH Oxidase Activation Circuit Is Required for Rapid Defense Signal Propagation. Proc Natl Acad Sci USA 110(21):8744–8749. 10.1073/pnas.1221294110 23650383 PMC3666735

[CR6] Egan MJ, Wang ZY, Jones MA, Smirnoff N, Talbot NJ (2007) Generation of reactive oxygen species by fungal NADPH oxidases is required for rice blast disease. Proc Natl Acad Sci U S A 104(28):11772–11777. 10.1073/pnas.0700574104 17600089 PMC1913907

[CR7] Elstner EF, Heupel A (1976) Inhibition of nitrite formation from hydroxylammoniumchloride: a simple assay for superoxide dismutase. Anal Biochem 70(20):616–620. 10.1016/0003-2697(76)90488-7 817618

[CR74] Engler C, Kandzia R, Marillonnet S (2008) A one pot, one step, precision cloning method with high throughput capability. PLoS One 3:e3647. 10.1371/journal.pone.0003647 PMC2574415 18985154

[CR8] Fang SM, Hou X, Liang XL (2021) Response mechanisms of plants under saline-alkali stress. Front Plant Sci 12:667458. 10.3389/fpls.2021.667458 34149764 PMC8213028

[CR9] Feng ZH, Liu XL, Jiang CJ, Liang ZW (2016) Comprehensive evaluation of rice (Oryza sativa japonica) germplasm for alkaline/saline tolerance at germination stage from Jilin Province, China. Soils Crops 5(2):120–127. 10.11689/j.issn.2095-2961.2016.02.009

[CR10] Feng ZH, Xu Y, Xie ZM et al (2024) Overexpression of abscisic acid biosynthesis gene OsNCED3 enhances survival rate and tolerance to alkaline stress in rice seedlings. Plants 13(12):1713. 10.3390/plants13121713 38931145 PMC11207436

[CR11] Garciadeblás B, Senn ME, Bañuelos MA, Rodríguez-Navarro A (2003) Sodium transport and HKT transporters: the rice model. Plant J 34(6):788–801. 10.1046/j.1365-313x.2003.01764.x 12795699

[CR12] Gestelen PV, Asard H, Caubergs RJ (1997) Solubilization and separation of a plant plasma membrane NADPH-O₂⁻ synthase from other NAD(P)H oxidoreductases. Plant Physiol 115(2):543–550. 10.1104/pp.115.2.543 12223822 PMC158513

[CR13] Glyan’Ko AK, Ischenko AA (2010) Structural and functional characteristics of plant NADPH oxidase: a review. Appl Biochem Microbiol 46(5):463–471. 10.1134/S0003683810050017 21061597

[CR14] Guan QJ, Liao X, He ML, Li XF, Wang ZY, Ma HY, Yu S, Liu SK (2017) Tolerance analysis of chloroplast OsCu/Zn-SOD overexpressing rice under NaCl and NaHCO₃ stress. PLoS ONE 12(10):e0186052. 10.1371/journal.pone.0186052 29020034 PMC5636109

[CR15] Guo SQ, Chen YX, Ju YL et al (2025) Fine-tuning gibberellin improves rice alkali-thermal tolerance and yield. Nature 639(8053):162–171. 10.1038/s41586-024-08486-7 39880957

[CR16] He C, Liu H, Chen D, Xie WZ, Wang M, Li Y, Gong X, Yan WH, Chen LL (2021) CRISPR-Cereal: a guide RNA design tool integrating regulome and genomic variation for wheat, maize and rice. Plant Biotechnol J 19(11):2141–2143. 10.1111/pbi.13645 34310056 PMC8541771

[CR17] Heath RL, Packer L (1968) Photoperoxidation in isolated chloroplasts. I. Kinetics and stoichiometry of fatty acid peroxidation. Arch Biochem Biophys 125(1):189–198. 10.1016/0003-9861(68)90654-1 5655425

[CR18] Hu CH, Wang PQ, Zhang PP, Nie XM, Li BB, Tai L, Liu WT, Li WQ, Chen KM (2020) NADPH Oxidases: The Vital Performers and Center Hubs during Plant Growth and Signaling. Cells 9(2):437. 10.3390/cells9020437 32069961 PMC7072856

[CR19] Jian L, Jun Z, Da X, Bozza PT (2012) Phosphatidylinositol 3-Kinase Plays a Vital Role in Regulation of Rice Seed Vigor via Altering NADPH Oxidase Activity. PLoS ONE 7(3):e33817. 10.1371/journal.pone.0033817 22448275 PMC3309022

[CR20] Jiang CJ, Liang ZW, Xie XZ (2023) Priming for saline-alkaline tolerance in rice: current knowledge and future challenges. Rice Sci 30(5):417–425. 10.1016/j.rsci.2023.05.003

[CR21] Junghe H, Ki-Hong J, Choon-Hwan L, Gynheung A (2004) Stress-inducible OsP5CS2 gene is essential for salt and cold tolerance in rice. Plant Sci 167(3):417–426. 10.1016/j.plantsci.2004.04.009

[CR22] Kader MA, Seidel T, Golldack D, Lindberg S (2006) Expressions of OsHKT1, OsHKT2, and OsVHA are differentially regulated under NaCl stress in salt-sensitive and salt-tolerant rice (Oryza sativa L.) cultivars. J Exp Bot 57(15):4257–4268. 10.1093/jxb/erl199 17088362

[CR23] Kerchev P, De Smet B, Waszczak C, Messens J, Breusegem FV (2015) Redox strategies for crop improvement. Antioxid Redox Signal 23(14):1186–1205. 10.1089/ars.2014.6033 26062101

[CR24] Li M, Kim C (2021) Chloroplast ROS and stress signaling. Plant Commun 3(1):100264. 10.1016/j.xplc.2021.100264 35059631 PMC8760138

[CR26] Liu J, Zhou J, Xing D (2012) Phosphatidylinositol 3-kinase plays a vital role in regulation of rice seed vigor via altering NADPH oxidase activity. PLoS ONE 7(3):e33817. 10.1371/journal.pone.0033817 22448275 PMC3309022

[CR28] Liu QY, He HH, Hu LF (2013) Research progress in plant Rboh genes function and activity regulation mechanism. Biotechnol Bull 32(11):8–13. 10.13560/j.cnki.biotech.bull.1985.2013.11.027

[CR25] Liu HB, Wang XD, Zhang YY, Dong JJ, Ma C, Chen WL (2015) NADPH Oxidase Rbohd Contributes to Autophagy and Hypersensitive Cell Death During the Plant Defense Response in Arabidopsis Thaliana. Biol Plant 59:570–580. 10.1007/s10535-015-0519-9

[CR73] Liu H, Ding Y, Zhou Y, Jin W, Xie K, Chen LL (2017) CRISPR-P 2.0: an improved CRISPR-Cas9 tool for genome editing in plants. Mol Plant 10(3):530–532. 10.1016/j.molp.2017.01.003 28089950

[CR31] Liu XL, Jin YY, Zhang H et al (2019) Abscisic acid primes rice seedlings for enhanced tolerance to alkaline stress by upregulating antioxidant defense and stress tolerance-related genes. Plant Soil 438(1):39–55. 10.1007/s11104-019-03992-4

[CR27] Liu MM, Yu HY, Ouyang B, Shi CM, Demidchik V, Hao ZF, Yu M, Shabala S (2020) NADPH oxidases and the evolution of plant salinity tolerance. Plant Cell Environ 43(12):2957–2968. 10.1111/pce.13869 33043459

[CR34] Liu XL, Xu C, Yang HT et al (2021) Root architectural and physiological responses in contrasting rice genotypes to saline-alkaline stress. Int J Agric Biol 26:401–410. 10.17957/IJAB/15.1849

[CR30] Liu XL, Ji P, Yang HT et al (2022a) Priming effect of exogenous ABA on heat stress tolerance in rice seedlings is associated with the upregulation of antioxidative defense capability and heat shock related genes. Plant Growth Regul 98(1):23–38. 10.1007/s10725-022-00828-7

[CR33] Liu XL, Xie XZ, Zheng CK, Wei LX, Li XW, Jin YY, Zhang GH, Jiang CJ, Liang ZW (2022b) RNAi-mediated suppression of the abscisic acid catabolism gene OsABA8ox1 increases abscisic acid content and tolerance to saline-alkaline stress in rice (Oryza sativa L). Crop J 10(2):354–367. 10.1016/j.cj.2021.06.011

[CR32] Liu XL, Liao JP, Zhong X, Duan XM, Hu YX, Liu JC, Liu ZK, Yang HT (2023a) Expression pattern of the OsRboh Family genes in rice under heat stress of different growth stages. Acta Agric Boreali-Sinica 38(5):1–8. 10.7668/hbnxb.20193902

[CR35] Liu XL, Zhong X, Liao JP et al (2023b) Exogenous abscisic acid improves grain filling capacity under heat stress by enhancing antioxidative defense capability in rice. BMC Plant Biol 23:619. 10.1186/s12870-023-04638-5 38057725 PMC10699063

[CR29] Liu XL, Ji P, Liao JP et al (2025) CRISPR/Cas knockout of the NADPH oxidase gene OsRbohB reduces ROS overaccumulation and enhances heat stress tolerance in rice. Plant Biotechnol J 23(2):335–361. 10.1111/pbi.14500 PMC11772341 39485884

[CR36] Livak KJ, Schmittgen TD (2001) Analysis of relative gene expression data using real-time quantitative PCR and the 2^–∆∆CT^ method. Methods 25:402–408. 10.1006/meth.2001.1262 11846609

[CR37] Lv BS, Li XW, Ma HY, Sun Y, Wei LX, Jiang CJ, Liang ZW (2013) Differences in growth and physiology of rice in response to different saline-alkaline stress factors. Agron J 105(6):1119–1128. 10.2134/agronj2013.0017er

[CR39] Ma X, Zhang Q, Zhu Q et al (2015) A robust CRISPR/Cas9 system for convenient, high-efficiency multiplex genome editing in monocot and dicot plants. Mol Plant 8(8):1274–1284. 10.1016/j.molp.2015.04.007 25917172

[CR38] Ma CK, Li Q, Song ZX, Su LJ, Tao WH, Zhou BB, Wang QJ (2022) Irrigation with magnetized water alleviates the harmful effect of saline-alkaline stress on rice seedlings. Int J Mol Sci 23(17):10048. 10.3390/ijms231710048 36077438 PMC9456538

[CR40] Meng F, Xiang D, Bu Z et al (2025) H₂O₂-mediated oxidation of PHOSPHATE STARVATION RESPONSE2 promotes adaptation to low phosphate in rice. Nat Commun 16(1):8760. 10.1038/s41467-025-63841-0 41034194 PMC12489096

[CR41] Mondal K, Singh RK, Prasad M, Dey N (2024) Newly identified Pijx gene: a weapon against both seedling and panicle blast in rice. Plant Cell Rep 43(4):105. 10.1007/s00299-024-03198-8 38522062

[CR42] Munns R, Tester M (2008) Mechanisms of salinity tolerance. Annu Rev Plant Biol 59:651–681. 10.1146/annurev.arplant.59.032607.092911 18444910

[CR43] Ndudzo A, Makuvise AS, Moyo S, Bobo ED (2024) CRISPR-Cas9 genome editing in crop breeding for climate change resilience: implications for smallholder farmers in Africa. J Agric Food Res 16:101132. 10.1016/j.jafr.2024.101132

[CR44] Nestler J, Liu S, Wen TJ et al (2014) Roothairless5, which functions in maize (Zea mays L.) root hair initiation and elongation encodes a monocot-specific NADPH oxidase. Plant J 79(5):729–740. 10.1111/tpj.12578 24902980

[CR45] Qi Y, Xie Y, Ge M et al (2024) Alkaline tolerance in plants: the AT1 gene and beyond. J Plant Physiol 303:154373. 10.1016/j.jplph.2024.154373 39454297

[CR46] Sagi M, Fluhr R (2006) Production of reactive oxygen species by plant NADPH oxidases. Plant Physiol 141(2):336–340. 10.1104/pp.106.078089 16760484 PMC1475462

[CR47] Seck PA, Diagne A, Mohanty S, Wopereis MCS (2012) Crops that feed the world 7: rice. Food Secur 4(1):7–24. 10.1007/s12571-012-0168-1

[CR48] Shen T, Yan RJ, Xu FJ, Wang QW, Chen D, Li KY, Ni L, Jiang MY (2023) The NADPH oxidase OsRbohD and OsRbohH negatively regulate saline-alkaline tolerance in rice. Environ Exp Bot 213:105445. 10.1016/j.envexpbot.2023.105445

[CR49] Shi Y, Chang YL, Wu HT, Shalmani A, Liu WT, Li WQ, Xu JW, Chen KM (2020) OsRbohB-mediated ROS production plays a crucial role in drought stress tolerance of rice. Plant Cell Rep 39(12):1767–1784. 10.1007/s00299-020-02603-2 32980968

[CR50] Siriporn S, Pongsathorn K, Bongkoj B, Thapana B, Tadao A, Hongya G, Teerapong B, Supachitra C (2013) Exogenous ABA induces salt tolerance in indica rice (Oryza sativa L.): the role of OsP5CS1 and OsP5CR gene expression during salt stress. Environ Exp Bot 86:94–105. 10.1016/j.envexpbot.2010.01.009

[CR51] Sun JK, He L, Li T (2019) Response of seedling growth and physiology of Sorghum bicolor (L.) Moench to saline-alkali stress. PLoS ONE 14:e0220340. 10.1371/journal.pone.0220340 31361760 PMC6667135

[CR52] Toki S, Hara N, Ono K, Onodera H, Tagiri A, Oka S, Tanaka H (2006) Early infection of scutellum tissue with Agrobacterium allows highspeed transformation of rice. Plant J 47(6):969–976. 10.1111/j.1365-313X.2006.02836.x 16961734

[CR53] Torres MA, Dangl JL, Jones JDG (2002) Arabidopsis gp91(phox) homologues AtrbohD and AtrbohF are required for accumulation of reactive oxygen intermediates in the plant defense response. Proc Natl Acad Sci U S A 99(1):517–522. 10.1073/pnas.012452499 11756663 PMC117592

[CR54] Wang GF, Li WQ, Li WY, Wu GL, Zhou CY, Chen KM (2013) Characterization of rice NADPH oxidase genes and their expression under various environmental conditions. Int J Mol Sci 14(5):9440–9458. 10.3390/ijms14059440 23629674 PMC3676792

[CR57] Wang MM, Yang F, Ma HY et al (2016a) Cooperative effects of sand application and flushing during the sensitive stages of rice on its yield in a hard saline-sodic soil. Plant Prod Sci 19(4):1–11. 10.1080/1343943X.2016.1195696

[CR58] Wang X, Zhang MM, Wang YJ, Gao YT, Li R, Wang GF, Li WQ, Chen KM (2016b) The plasma membrane NADPH oxidase OsRbohA plays a crucial role in developmental regulation and drought-stress response in rice. Physiol Plant 156(4):421–443. 10.1111/ppl.12489 26400148

[CR56] Wang MM, Rengasamy P, Wang ZC et al (2018) Identification of the most limiting factor for rice yield using soil data collected before planting and during the reproductive stage. Land Degrad Dev 29:2310–2320. 10.1002/ldr.3026

[CR55] Wang HQ, Zhao XY, Xuan W, Wang P, Zhao FJ (2023) Rice roots avoid asymmetric heavy metal and salinity stress via an RBOH-ROS-auxin signaling cascade. Mol Plant 16(10):1678–1694. 10.1016/j.molp.2023.09.007 37735869

[CR60] Wei LX, Lv BS, Wang MM, Ma HY, Yang HY, Liu XL, Jiang CJ, Liang ZW (2015) Priming effect of abscisic acid on alkaline stress tolerance in rice (Oryza sativa L.) seedlings. Plant Physiol Biochem 90:50–57. 10.1016/j.plaphy.2015.03.002 25780993

[CR59] Wei LX, Lv BS, Li XW et al (2017) Priming of rice (Oryza sativa L.) seedlings with abscisic acid enhances seedling survival, plant growth, and grain yield in saline-alkaline paddy fields. Field Crops Res 203:86–93. 10.1016/j.fcr.2016.12.024

[CR61] Wellburn A, Lichtenthaler H (1984) Formulae and program to determine total carotenoids and chlorophylls A and B of leaf extracts in different solvents. Adv Photosynth Res 2:9–12

[CR62] Wong HL, Pinontoan R, Hayashi K, Tabata R, Yaeno T, Hasegawa K, Kojima C, Yoshioka H, Iba K, Kawasaki T (2007) Regulation of Rice NADPH Oxidase by Binding of Rac GTPase to Its N-Terminal Extension. Plant Cell 19(12):4022–4034. 10.1105/tpc.107.055624 18156215 PMC2217649

[CR63] Xiao N, Wu Y, Zhang X et al (2024) Pijx confers broad-spectrum seedling and panicle blast resistance by promoting the degradation of ATP β subunit and OsRbohC-mediated ROS burst in rice. Mol Plant 16(11):672–675. 10.1016/j.molp.2023.10.001 38401544

[CR64] Xie X, Ma X, Zhu Q, Zeng D, Li G, Liu YG (2017) CRISPR-GE: a convenient software toolkit for CRISPR-Based genome editing. Mol Plant 10(9):1246–1249. 10.1016/j.molp.2017.06.004 28624544

[CR65] Xu Y, Feng ZH, Lu GR et al (2025) Transcriptome analysis of OsNCED3 transgenic rice reveals the response mechanism to alkaline stress. Plant Physiol Biochem 228:110279. 10.1016/j.plaphy.2025.110279 40749471

[CR66] Yamauchi T, Yoshioka M, Fukazawa A et al (2017) An NADPH Oxidase RBOH Functions in Rice Roots during Lysigenous Aerenchyma Formation under Oxygen-Deficient Conditions. Plant Cell 29(4):775–790. 10.1105/tpc.16.00976 28351990 PMC5435434

[CR67] Yoshiaki Y, Kazunori G, Ryota T, Iwano M, Takayama S, Lsogai A, Che FS (2005) Function of the rice gp91^phox^ homologs OsrbohA and OsrbohE genes in ROS-dependent plant immune responses. Plant Biotechnol 22(2):127–135. 10.5511/plantbiotechnology.22.127

[CR68] Yoshioka H, Sugie K, Park HJ, Maeda H, Tsuda N, Kawakita K, Doke N (2001) Induction of Plant gp91 phox Homolog by Fungal Cell Wall, Arachidonic Acid, and Salicylic Acid in Potato. Mol Plant Microbe Interact 14(6):725–736. 10.1094/MPMI.2001.14.6.725 11386368

[CR69] Zhang D, Chen L, Li D, Lv B, Chen Y, Chen J, Yan XJ, Liang JS (2014) OsRACK1 is involved in abscisic acid- and H₂O₂-mediated signaling to regulate seed germination in rice (Oryza sativa, L). PLoS ONE 9(5):e97120. 10.1371/journal.pone.0097120 24865690 PMC4035261

[CR70] Zhang H, Liu XL, Zhang RX et al (2017) Root damage under alkaline stress is associated with reactive oxygen species accumulation in rice (Oryza sativa L). Front Plant Sci 8:1580. 10.3389/fpls.2017.01580 28943882 PMC5596797

[CR71] Zhang Y, Han E, Peng Y et al (2022) Rice co-expression network analysis identifies gene modules associated with agronomic traits. Plant Physiol 190(2):1526–1542. 10.1093/plphys/kiac339 35866684 PMC9516743

[CR72] Zhao L, Hu H, Chen J et al (2024) A 9.5-kb deletion in the 1st intron of OsMADS51 enhances temperature sensitivity in rice. Crop J 12(4):1–10. 10.1016/j.cj.2024.05.010

