# Supplementary material for: Knockout of OsRbohD (the NADPH Oxidase Gene) Enhances Saline-Alkaline Stress Tolerance and Grain Yield in Rice by Reducing ROS Accumulation
Source: Rice (N Y). 2026 Jun 23;19:59. doi: 10.1186/s12284-026-00928-2 (PMC13562477; doi:10.1186/s12284-026-00928-2)
Supplement: Supplementary file 1 [file 12284_2026_928_MOESM1_ESM.docx]

**Figure S1**

**Fig. S1** The *OsRbohD*-knockout (*OsRbohD*-KO) and *OsRbohD*-overexpression (*OsRbohD*-OE) vector construction. (a) Two gRNAs were set and constructed into the CRISPR-Cas9 vector. (b) *OsRbohD*-overexpression vector by homologous recombination method.

**Figure S2**

**Fig. S2** The changes in gene sequence of *OsRbohD*-KO rice lines and hygromycin resistance test of all the *OsRbohD*-KO and *OsRbohD*-OE rice plants. The hygromycin resistance test of the *OsRbohD*-KO mutational (a) and *OsRbohD*-OE rice lines (b). (c) Genomic DNA tests of the *OsRbohD*-KO lines (KO-7 and KO-15) rice compared with the WT plants. (d) Standard PCR for the genotyping assay of the different rice lines. (e) The hygromycin resistance test of the different rice lines. (f) Cas9 test of the different rice lines. PC: positive control; WT: the WT plants; PW: control PCR reaction mixture (primers and double-distilled H_2_O).

**Figure S3**

**Fig. S3** (a) Relative gene expression levels of *OsRbohD* under DPI and PCMB treatments. The expression levels of the CK treatment were set as the unit to calculate the expression levels for other treatments. (b) Relative gene expression levels of O*sRbohD* in the T2 rice seedlings of different rice lines. Three gene specific primers were designed using Primer 5.0 software from the 4^th^ exon to the 8^th^ exon outside the gene-editing zone. The expression levels of the WT plants were set as the unit to calculate the expression levels. Relative gene expression levels of other *OsRboh* family genes of different rice lines under CK (c), SS (d) and AS (e) conditions. The expression levels of the WT plants were set as the unit to calculate the expression levels for each gene. qRT-PCR was performed using *OsACT1* as an internal standard. Data are expressed as mean ± SD, *n=3*. Different letters on the columns represent significant differences (*P*<0.05) between different rice lines or different treatments based on Duncan’s test.

**Figure S4**

**Fig. S4** The growth morphologies of *OsRbohD*-KO, WT and *OsRbohD*-OE plants. The growth morphologies of *OsRbohD*-KO (KO-7 and KO-15), WT and *OsRbohD*-OE (OE-3 and OE-1) plants were photographed at the (a) seedling, (b) tillering, (c) heading and (d) filling stages.

**Figure S5**

**Fig. S5** Germination status of *OsRbohD*-KO, WT and *OsRbohD*-OE plants under saline and alkaline stress conditions. The germination status of *OsRbohD*-KO lines (KO-7, KO-15), WT plants, and *OsRbohD*-OE lines (OE-3, OE-1) were photographed at the 7^th^ day under different treatments.

**Figure S6**

**Fig. S6** Seedlings growth status of *OsRbohD*-KO, WT and *OsRbohD*-OE plants under salt and alkaline stress conditions. Photographs of seedlings growth status of *OsRbohD*-KO lines (KO-7,KO-15), WT plants, and *OsRbohD*-OE lines (OE-3, OE-1) were taken at the 3^rd^ day of (a) M-SS1, (b) M-SS2, (c) M-SS3, (d) M-AS1, (e) M-AS2, (f) M-AS3, (g) CK2 treatments.

**Figure S7**

**Fig. S7** Seedlings growth status of *OsRbohD*-KO, WT and *OsRbohD*-OE plants under salt and alkaline stress conditions. Photographs of seedlings growth status of *OsRbohD*-KO lines (KO-7, KO-15), WT plants, and *OsRbohD*-OE lines (OE-3, OE-1) were taken at the 7^th^ day of (a) M-SS1, (b) M-SS2, (c) M-SS3, (d) M-AS1, (e) M-AS2, (f) M-AS3, (g) CK2 treatments.

**Figure S8**

**Fig. S8** MDA and MI in rice leaves of *OsRbohD*-KO, WT and *OsRbohD*-OE plants under salt and alkaline stress conditions. (a) MDA contents and (b) MI in rice leaves of *OsRbohD*-KO lines (KO-7 and KO-15), WT plants, and *OsRbohD*-OE lines (OE-3 and OE-1) were measured at the 7^th^ day of different treatments. Data are expressed as mean ± SD, *n=5*. Different letters on the columns represent significant differences (*P*<0.05) between different rice lines based on Duncan’s test.

**Figure S9**

**Fig. S9** Seedlings growth status of *OsRbohD*-KO, WT and *OsRbohD*-OE plants under saline and alkaline stress conditions. Photographs of seedlings growth status of *OsRbohD*-KO lines (KO-7, KO-15), WT plants, and *OsRbohD*-OE lines (OE-3, OE-1) were taken on the 6^th^ day (a) and re-watering for 24 h (b) under different saline (M-SS1, M-SS2, M-SS3) and alkaline (M-AS1, M-AS2, M-AS3) treatments.

**Figure S10**

**Fig. S10** Seedlings growth status of *OsRbohD*-KO, WT and *OsRbohD*-OE plants under salt and alkaline stress conditions. Leaf withering rates (a, b) and chlorophyll content (c, d) were measured on the 6^th^ day and re-watering for 24 h under different saline (M-SS1, M-SS2, M-SS3) and alkaline (M-AS1, M-AS2, M-AS3) treatments. The data are expressed as the mean ± SD; *n=5*. Different letters on the columns represent significant differences (*P<0.05*) between different lines of the same treatment based on Duncan’s test.

**Figure S11**

**Fig. S11** Na^+^, K^+^ and mineral element content in rice leaves of *OsRbohD*-KO, WT and *OsRbohD*-OE plants under salt and alkaline stress conditions. (a) Na^+^ contents, (b) K^+^ contents, (c) ratio of Na^+^/K^+^, (d) Fe contents, (e) Mn contents and (f) Zn contents in rice leaves of *OsRbohD*-KO lines (KO-7 and KO-15), WT plants, and *OsRbohD*-OE lines (OE-3 and OE-1) were measured at the 7^th^ day of different treatments. Data are expressed as mean ± SD, *n=5*. Different letters on the columns represent significant differences (*P*<0.05) between different rice lines based on Duncan’s test.

**Figure S12**

**Fig. S12** Seedlings growth status of *OsRbohD*-KO, WT and *OsRbohD*-OE plants under salt-alkaline soil conditions. Photographs of seedlings growth status of *OsRbohD*-KO lines (KO-7, KO-15), WT plants, and *OsRbohD*-OE lines (OE-3, OE-1) were taken at the 10^th^ day of different treatments (a-j).

**Figure S13**

**Fig. S13** Plants growth status of *OsRbohD*-KO, WT and *OsRbohD*-OE plants under different degree of salt-alkaline soil conditions. (a) CK3: normal soils with the pH of 7.53; (b) YS2: saline-alkaline soil with the pH of 8.52; (c) YS5: saline-alkaline soil with the pH of 9.33; (d) YS7: saline-alkaline soil with the pH of 9.78. Photographs of plants growth status of *OsRbohD*-KO lines (KO-7, KO-15), WT plants, and *OsRbohD*-OE lines (OE-3, OE-1) were taken at tillering stage of different treatments (a-d).

**Figure S14**

**Fig. S14** Plants growth status of *OsRbohD*-KO, WT and *OsRbohD*-OE plants under different degree of salt-alkaline soil conditions. (a) CK3: normal soils with the pH of 7.53; (b) YS2: saline-alkaline soil with the pH of 8.52; (c) YS5: saline-alkaline soil with the pH of 9.33; (d) YS7: saline-alkaline soil with the pH of 9.78. Photographs of plants growth status of *OsRbohD*-KO lines (KO-7, KO-15), WT plants, and *OsRbohD*-OE lines (OE-3, OE-1) were taken at heading stage of different treatments (a-d).

**Figure S15**

**Fig. S15** Plants growth status of *OsRbohD*-KO, WT and *OsRbohD*-OE plants under different degree of salt-alkaline soil conditions. (a) CK3: normal soils with the pH of 7.53; (b) YS2: saline-alkaline soil with the pH of 8.52; (c) YS5: saline-alkaline soil with the pH of 9.33; (d) YS7: saline-alkaline soil with the pH of 9.78. Photographs of plants growth status of *OsRbohD*-KO lines (KO-7, KO-15), WT plants, and *OsRbohD*-OE lines (OE-3, OE-1) were taken at filling stage of different treatments (a-d).

**Figure S16**

**Fig. S16** Plant phenotypes of *OsRbohD*-KO, WT and *OsRbohD*-OE plants at mature stage under different degree of salt-alkaline soil conditions. (a) CK3: normal soils with the pH of 7.53; (b) YS2: saline-alkaline soil with the pH of 8.52; (c) YS5: saline-alkaline soil with the pH of 9.33; (d) YS7: saline-alkaline soil with the pH of 9.78. Photographs of plants phenotypes of *OsRbohD*-KO lines (KO-7, KO-15), WT plants, and *OsRbohD*-OE lines (OE-3, OE-1) were taken at mature stage.

**Figure S17**

**Fig. S17** Panicle phenotype of *OsRbohD*-KO, WT and *OsRbohD*-OE plants at mature stage under different degree of salt-alkaline soil conditions. (a) CK3: normal soils with the pH of 7.53; (b) YS2: saline-alkaline soil with the pH of 8.52; (c) YS5: saline-alkaline soil with the pH of 9.33; (d) YS7: saline-alkaline soil with the pH of 9.78. Photographs of panicle phenotypes of *OsRbohD*-KO lines (KO-7, KO-15), WT plants, and *OsRbohD*-OE lines (OE-3, OE-1) were taken at mature stage.

**Figure S18**

**Fig. S18** Plant growth indexes of *OsRbohD*-KO, WT and *OsRbohD*-OE plants at mature stage under different treatments. CK3: normal soils with the pH of 7.53; YS2: saline-alkaline soil with the pH of 8.52; YS5: saline-alkaline soil with the pH of 9.33; YS7: saline-alkaline soil with the pH of 9.78. (a) Shoot length (SL), (b) shoot dry weight (SDW), (c) primary branches (PB), (d) secondary branches (SB), (e) panicle length (PL) and (f) panicle weight (PW) were measured at the mature stage. Data are expressed as mean ± SD, *n=5*. Different letters on the columns represent significant differences (*P*<0.05) between different lines of the same treatment based on Duncan’s test.

**Figure S19**

**Fig. S19** Root growth status of *OsRbohD*-KO, WT and *OsRbohD*-OE plants at mature stage under different degree of salt-alkaline soil conditions. (a) CK3: normal soils with the pH of 7.53; (b) YS2: saline-alkaline soil with the pH of 8.52; (c) YS5: saline-alkaline soil with the pH of 9.33; (d) YS7: saline-alkaline soil with the pH of 9.78. Photographs of root status of *OsRbohD*-KO lines (KO-7, KO-15), WT plants, and *OsRbohD*-OE lines (OE-3, OE-1) were taken at mature stage (a-d). (e) Main root length, (f) root dry weight were measured at mature stage.

**Table S1 Genes and primer sequences used in this study.**

| **Purpose** | **Name** | **Primers sequences (F: Forward R: Reverse)** |
| --- | --- | --- |
| Primers for *OsRbohD*-OE vector | *OsRbohD-*PCR | F: Actag ggtctcG CACC ATGAGGGCGGGAATCGGCAGCG |
|  |  | R: Actag ggtctcT ACCG TTAGAAATGCTCCTTGTGAAACTCAAATTTTGACG |
|  | OHD-JJF (for colony PCR test) | CAATAACTTCGGCACCTGGG |
|  | NOS-R (for colony PCR test) | ATCATCGCAAGACCGGCAAC |
| Primers for *OsRbohD*-KO vector | sgRNA-1 | GCATTGCGGGAAATCGTCGC GGG |
|  | sgRNA-2 | AGCTGTTCGACACCTTGAGC CGG |
|  | Cas-9-gRT1 | CATTGCGGGAAATCGTCGCgttttagagctagaaat |
|  | Cas-9-OsU6a | GCGACGATTTCCCGCAATGCggcagccaagccagca |
|  | Cas-9-gRT2 | AGCTGTTCGACACCTTGAGCgttttagagctagaaat |
|  | Cas-9-OsU6b | GCTCAAGGTGTCGAACAGCTCaacacaagcggcagc |
|  | U-F | CTCCGTTTTACCTGTGGAATCG |
|  | gRNA-R | CGGAGGAAAATTCCATCCAC |
|  | Pps-R | TTCAGAggtctcTaccgACTAGTATGGAATCGGCAGCAAAGG |
|  | Pgs-2 | AGCGTGggtctcGtcagggTCCATCCACTCCAAGCTC |
|  | Pps-2 | TTCAGAggtctcTctgacacTGGAATCGGCAGCAAAGG |
|  | Pgs-L | AGCGTGggtctcG ctcg ACGCGTATCCATCCACTCCAAGCTC |
|  | SP-L (for colony PCR test) | GCGGTGTCATCTATGTTACTAG |
|  | SP-R (for colony PCR test) | TGCAATAACTTCGTATAGGC |
| Primers for test | HYG-test | F: AGATGATCGCCAAGTCCGAGC |
|  |  | R: CTTGATGATGAGGTCCTTCTTG |
|  | Cas9 test | F: CCGGATGCCTCCGCTCGAAGTA |
|  |  | R: GCCGTGCACAGGGTGTCACGTT |
|  | Standard PCR | F: CTTCATCAGCGGCCCCAACA |
|  |  | R: GCGCGAGTCGAAGCTGTTGT |
|  | *OsRbohD*-primer-1 (qRT-PCR) | F: GTCAAAGGAGTGGAGGGCATAAC |
|  |  | R: CCAGGCTACGGCGGAACC |
|  | *OsRbohD*-primer-2 (qRT-PCR) | F: TATGCCGCAACACCATCACT |
|  |  | R: ACACCAACCACAATTGCTGC |
|  | *OsRbohD*-primer-3 (qRT-PCR) | F: AGGTGGCCATCTATCCTGGT |
|  |  | R: CCCCAGGTGCCGAAGTTATT |
| Primers for qRT-PCR | *OsACT1*  (Os03g0718100) | F: CATTGGTGCTGAGCGTTTCC |
|  |  | R: AGAAACAAGCAGGAGGACGG |
|  | *OsBI-1*  (Os02g0125300) | F: AGCACGCACTCACCCTCTTC |
|  |  | R: TTCCTCTTCTTCTCCTCCGACTTG |
|  | *OsKOD-1*  (Os04g0507950) | F: GCAAGTCACAAAACGGCAATGG |
|  |  | R: CACCTGATACAACTGGAAGGATGG |
|  | *OsPEX11*  (Os03g0302000) | F: GCTGGAAGAGATCGGCAAACTG |
|  |  | R: GTCCTCACCACATCGCTCTCC |
|  | *OsJRL*  (Os01g0348900) | F: GGGCGGAAATGGAGGGTCAG |
|  |  | R: ACACCGATGTAGTTGAAGGCAATG |
|  | *OsHKT1*  (Os06g0701700) | F: CGCTGGCTTTCTGCTGGTTC |
|  |  | R: AGGAGACTGTGACCGAGAATGAG |
|  | *OsHKT7*  (Os04g0607600) | F: TCACAACTCTCCTACCTGACCATC |
|  |  | R: CACTGACAACTTCGACAACAATGC |
|  | *OsP5CS1*  (Os05g0455500) | F: GGGGCGACAGCGACTTAGG |
|  |  | R: GACCAACAGCGGCACAAGC |
|  | *OsP5CS2*  (Os01g0848200) | F: CCTCTGGTGCTGTTGGTGTTG |
|  |  | R: TCCCGTCCATGTCCATCTGTG |
|  | *OsRbohH*  (Os12g0541300) | F: CACCACCTCTTCGTCATCGTCTAC |
|  |  | R: TGTCGTCTGCTTCCACCAAGTC |
|  | *OsRbohI*  (Os11g0537400) | F: GTGGCGGAGACGGACAAGAAG |
|  |  | R: TTGGCGTGGTTGAGCGACTG |
|  | *OsRbohB*  (Os09g0438000) | F: TCAACAGCAAGTAGCAACAAGAAGAG |
|  |  | R: TCCATTTCAGCAACCTCATTCATCAC |
|  | *OsRbohF*  (Os08g0453700) | F: ACCTCCTCATCGTCGTCTACCTC |
|  |  | R: GTCGTCCTCTGGTGCCACTTG |
|  | *OsRbohG*  (Os05g0465800) | F: ATGCTGCAACCTACGCCTCAC |
|  |  | R: GCTGTCACCATACCACGGAGTAG |
|  | *OsRbohD*  (Os05g0528000) | F: GCTACTATCTGGAGGACAACTGGAAG |
|  |  | R: ATCGGTGGCGGTATTGTATGAATTTC |
|  | *OsRbohE*  (Os01g0835500) | F: GAGGAGATGACCGACCAGAACTTC |
|  |  | R: CGCCGACGCACTCAGTATAATAAC |
|  | *OsRbohA*  (Os01g0734200) | F: ACCTCTCCCGCTCCGACTTC |
|  |  | R: CCTTGTTAATCGTGTCCACCTTCATC |
|  | *OsRbohC*  (Os01g0360200) | F: GACCCATTTCGCACGACCTAATTG |
|  |  | R: ACAGGCTCACCACAGTAGAACAC |
